# Effects of community youth teams facilitating participatory adolescent groups, youth leadership activities and livelihood promotion to improve school attendance, dietary diversity and mental health among adolescent girls in rural eastern India (JIAH trial): A cluster-randomised controlled trial

**DOI:** 10.1016/j.ssmph.2022.101330

**Published:** 2022-12-27

**Authors:** Komal Bhatia, Suchitra Rath, Hemanta Pradhan, Subhashree Samal, Andrew Copas, Sumitra Gagrai, Shibanand Rath, Raj Kumar Gope, Nirmala Nair, Prasanta Tripathy, Kelly Rose-Clarke, Audrey Prost

**Affiliations:** a Institute for Global Health, University College London, London, UK; b Ekjut, Chakradharpur, Jharkhand, India; c Department of Global Health and Social Medicine, King's College London, London, UK

**Keywords:** Adolescent health, India, Cluster randomised controlled trial, Mental health, Nutrition, Peer-facilitation

## Abstract

**Objectives:**

To evaluate whether and how community youth teams facilitating participatory adolescent groups, youth leadership and livelihood promotion improved school attendance, dietary diversity, and mental health among adolescent girls in rural India.

**Design:**

A parallel group, two-arm, superiority, cluster-randomised controlled trial with an embedded process evaluation.

**Setting, intervention and participants:**

38 clusters (19 intervention, 19 control) in West Singhbhum district in Jharkhand, India. The intervention included participatory adolescent groups and youth leadership for boys and girls aged 10–19 (intervention clusters only), and family-based livelihood promotion (intervention and control clusters) between June 2017 and March 2020. We surveyed 3324 adolescent girls aged 10–19 in 38 clusters at baseline, and 1478 in 29 clusters at endline. Four intervention and five control clusters were lost to follow up when the trial was suspended due to the COVID-19 pandemic. Adolescent boys were included in the process evaluation only.

**Primary and secondary outcome measures:**

Primary: school attendance, dietary diversity, and mental health; 12 secondary outcomes related to education, empowerment, experiences of violence, and sexual and reproductive health.

**Results:**

In intervention vs control clusters, mean dietary diversity score was 4·0 (SD 1·5) vs 3·6 (SD 1·2) (adjDiff 0·34; 95%CI -0·23, 0·93, p = 0·242); mean Brief Problem Monitor-Youth (mental health) score was 12·5 (SD 6·0) vs 11·9 (SD 5·9) (adjDiff 0·02, 95%CI -0·06, 0·13, p = 0·610); and school enrolment rates were 70% vs 63% (adjOR 1·39, 95%CI 0·89, 2·16, p = 0·142). Uptake of school-based entitlements was higher in intervention clusters (adjOR 2·01; 95%CI 1·11, 3·64, p = 0·020). Qualitative data showed that the community youth team had helped adolescents and their parents navigate school bureaucracy, facilitated re-enrolments, and supported access to entitlements. Overall intervention delivery was feasible, but positive impacts were likely undermined by household poverty.

**Conclusions:**

Participatory adolescent groups, leadership training and livelihood promotion delivered by a community youth team did not improve adolescent girls’ mental health, dietary diversity, or school attendance in rural India, but may have increased uptake of education-related entitlements.

**Trial registration:**

ISRCTN17206016

## Highlights

• Large cluster RCT of a community-based, peer-facilitated group intervention to improve adolescent health in rural India.
• No substantial effects found on girls' school attendance, dietary diversity, or mental health.
• Intervention group more likely to use school-related entitlements including cash stipends, uniforms, books.
• Adolescent boys had sustained engagement and comprised 40% of attendees at participatory groups.
• Household poverty was a barrier to securing the resources and support necessary for positive changes in primary outcomes.

## Author contributions

NN, SR, HP, SS, AP and KRC conceptualised the study. AC, HP, KB and KRC developed the statistical data analysis plan. KB, HP and SS conducted analyses. SR, SS, NN and SG trained the intervention team. HP, ShR, SS and SG trained the data collection team. SR, SS and NN supervised all study activities. SS and SR led the process evaluation. KB and AP wrote the first draft of the paper. KB and SR contributed equally to this paper. All authors critically reviewed, commented on, and revised drafts.

## 1 Introduction

At 252 million strong, India's adolescent cohort is the largest in the world (UNICEF. UNICEF Data Warehouse, 2020). Investing in adolescent health and wellbeing offers a triple dividend: benefits for the current generation of adolescents, for their health and wellbeing as adults, and for the next generation (Patton et al., 2016). Adolescent health and wellbeing are affected by drivers that cut across the life-course and across health, education, nutrition, and social protection sectors. As a result, there is a critical need for robust evaluations of intersectoral policies and programmes at structural, institutional, and community levels.

The Rashtriya Kishor Swasthya Karyakram (RKSK), India's 2014 flagship adolescent health promotion strategy, includes health facility- and community-based activities with a focus on adolescent participation and leadership. It includes provision for a peer educator intervention to increase adolescents' knowledge of health and build their skills and capacities to resolve their health concerns. RKSK peer educators are trained to facilitate adolescent groups and deliver a curriculum of topics related to sexual and reproductive health, nutrition, mental health, and violence. The RKSK peer education intervention is being implemented as a pilot in select geographies in India, but an intervention like RKSK's proposed curriculum has not yet been evaluated experimentally for its effects on health. To address this research gap, and following formative research including a global systematic review, qualitative study and cross-sectional survey (Rose-Clarke et al., 2019, Rose-Clarke, Bentley, Marston, & Prost, 2019), in 2017 we developed a peer-led intervention (the Jharkhand Initiative for Adolescent Health, or JIAH) to improve adolescent health building on RKSK priorities and using principles of participatory learning and action (PLA).

We offered the intervention to girls and boys aged 10–19 years in rural communities in the 50 villages of a district of the eastern Indian state of Jharkhand where the RKSK had not yet been implemented. We tested its effectiveness in a cluster-randomised controlled trial with an embedded mixed-methods process evaluation. Our formative research identified four focal areas that were important to adolescents and their families and might also be influenced through a community intervention: participation in education, undernutrition, mental health, and violence. These focal areas were reflected in the trial's outcomes, as described below. We aimed to evaluate whether community youth teams facilitating participatory peer-led groups, youth leadership activities and livelihood promotion could improve school attendance, dietary diversity, and mental health among adolescent girls in rural Jharkhand. Our process evaluation aimed to assess intervention implementation, context and mechanisms of impact.

## 2 Methods

This article adheres to 2010 Consolidated Standards of Reporting Trials (CONSORT) statement: extension to cluster randomised trials (Campbell et al., 2012) and the Template for Intervention Description and Replication (TIDieR) (Hoffmann et al., 2014) checklists (Supplementary Files S1 and S2, Tables S1 and S2).

### 2.1 Study design and setting

**Fig. 1:**
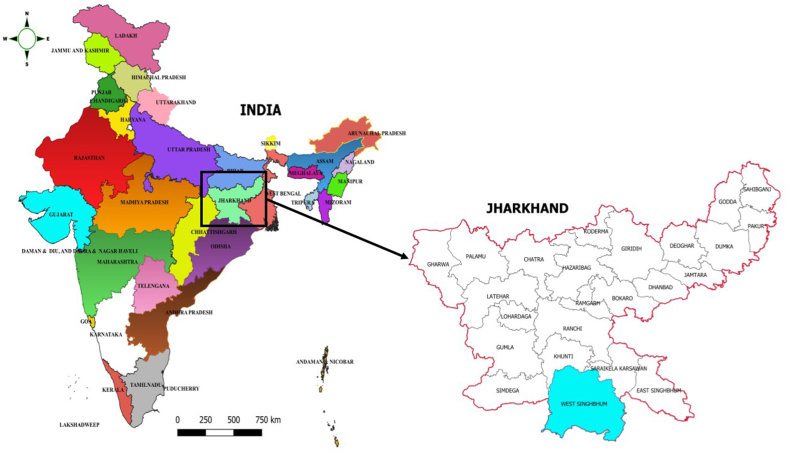
Location of study areas.

We conducted a parallel group, two-arm, superiority, cluster-randomised controlled trial with 1:1 allocation ratio, with cross-sectional surveys before and after the intervention. The trial was conducted in 38 clusters selected purposively from Khuntpani block in West Singhbhum district in Jharkhand (Fig. 1), each with a population of approximately 1000 (range, 723 to 1962) people living in the geographic area covering a village and its neighbouring hamlets. The 38 clusters included 50 villages and hamlets, and were separated by distinct natural boundaries (e.g. hills, water bodies) to prevent contamination. The study area included a population of approximately 40,000. We obtained written consent for participation of clusters from village leaders.

The intervention and data collection were led by Ekjut , an Indian civil society organisation, in collaboration with University College London. The implementation partner partitioned the intervention and data collection teams: they met on different days, and data monitors were led by a separate manager.

The published trial protocol (Rath et al., 2020) includes a discussion of ethical issues arising from the study, as well as details of the intervention's theory of change, process evaluation methods, and statistical analysis plans.

### 2.2 Study participants

#### 2.2.1 Survey participants

Individual survey participants were adolescent girls aged 10–19 residing in the study area. We excluded girls who ordinarily lived outside study clusters (for example, those studying or working away from home) during the baseline and endline surveys. Participants were interviewed separately about their health and education, and caregivers provided information on household socio-demographic characteristics.

Prior to the interview, data monitors visited each household in the study clusters to identify eligible adolescents, explain the objective of the interview and obtain consent. We sought written informed consent from adult caregivers for the household interview. Adolescents were asked for written consent for their participation; for adolescents under 18, we also sought consent from their caregiver. Adolescent consent superseded caregiver consent.

While the baseline and endline surveys were conducted in the same clusters, adolescent girls who were interviewed at the start of the intervention may or may not have been eligible or available for endline data collection. We did not link participants’ baseline and endline information as the evaluation was cross-sectional in design and we did not have consent for data linkage. Adolescent girls who met eligibility criteria for the surveys were invited to join the study regardless of their participation in intervention activities.

We excluded adolescent boys from surveys as we did not have the budget to extend surveys to them.

#### 2.2.2 Process evaluation participants

All adolescent boys and girls aged 10–19 and their parents or caregivers who took part in any intervention activity were eligible for all components of the process evaluation. Other participants included local health workers, schoolteachers and members of the community who engaged with the intervention. Members of the community youth team (*yuva saathi*, livelihood promotion coordinators and leadership coordinators) were also included as participants in small group discussions designed to understand contextual factors that shaped intervention delivery and mechanisms of impact.

Consent procedures for qualitative interviews and focus group discussions conducted during the process evaluation were identical to those for surveys.

### 2.3 Randomisation and masking

We randomised clusters within five strata based on availability of secondary school services and adolescent clubs run by the Integrated Development Federation, and population size: (1) a secondary school and an adolescent club, (2) a secondary school and no adolescent club, (3) an adolescent club but no secondary school, (4) neither a secondary school nor an adolescent club, and (5) population exceeding 1500. Randomisation was conducted on the 22^nd^ of February 2017 at a meeting attended by local community health workers, village leaders from enrolled clusters, and a government representative. After explaining the intervention and purpose of randomisation to meeting participants, the evaluation team members numbered clusters from 1 to 38 and displayed this allocation sequence on a wall. Cluster numbers were printed on identical balls which were then sorted into the five strata for random allocation. For each stratum, meeting participants inserted corresponding numbered balls into a tombola device and spun it around to sequentially allocate balls to the intervention and control arms. The randomisation meeting was video recorded, along with community representatives’ views on the process. Allocation was not concealed from participants of the randomisation meeting, study participants, or the intervention team due to the nature of the intervention. Data monitors were masked to allocation.

### 2.4 Procedures

We followed identical procedures for the baseline and endline surveys. A team of 12 female data monitors aged 20–24 living in or close to study clusters conducted the surveys. Data monitors received four days of training in interviewing skills, questionnaire administration and anthropometry, and participated in fortnightly review meetings. The trainers conducted a standardisation exercise with ten adolescent girls to assess Technical Error of Measurement which indicated high inter-observer coefficients of reliability (0·98 for height and 0·99 for weight and mid-upper arm circumference (MUAC)) prior to the baseline survey.

Survey interviews began with a caregiver questionnaire on household composition, socio-demographic status, economic activity and assets, and details of amenities in the home (e.g., kitchen garden, type of toilet). Data monitors asked adolescent girls about the language (Hindi, Ho, or Odia) they were most comfortable conversing in and used that to explain instructions and phrase questions. Adolescent girls responded to questions on education and literacy, general health and use of healthcare services, dietary intake using food list-based 24-h recall, mental health based on a checklist of symptoms, resilience and self-efficacy, experiences of violence, substance use, decision-making and gender norms, social support networks, and menstrual hygiene. Married adolescents and those aged 15–19 were asked about their knowledge of sexual and reproductive health. The survey instrument was modified at endline to include questions on exposure to the intervention and outcomes not assessed at baseline (see Supplementary File S3, Table S3). Data monitors measured adolescent girls’ height, weight and MUAC to assess anthropometric status. Data were collected using smartphones programmed with CommCare (Dimagi Corp.) for electronic data capture in Hindi and English. Automated skip constraints and consistency checks in CommCare were supplemented with routine and random quality checks to maintain data validity. Completed responses were downloaded from the server every week.

Process evaluation data were derived from two sources: (1) project documents and (2) qualitative interviews, case studies and small group discussions conducted by the community youth team, intervention supervisors, a process evaluation officer and process evaluation manager. Project documents included: registers listing the number and type of participants at each intervention activity; attendance logs tracking adolescents’ participation longitudinally within each adolescent group formed in a cluster; structured summaries of intervention activities and PLA processes to identify, prioritise and address adolescent health; minutes from review meetings and training exercises for the intervention team; and short case stories of participant engagement documented by the community youth team. The process evaluation manager conducted qualitative small group discussions (n=4) with the intervention team towards the end of the study to reflect on implementation delivery, context, and possible impact or barriers to success. Intervention supervisors collected qualitative data from participants in intervention clusters using individual (n = 13) and group (n = 3) case studies. For individual case studies, they conducted semi-structured interviews with 13 adolescents (six male, seven female) to ask about their engagement with the intervention, and separately sought the views of their parents and teachers on whether adolescents had been influenced by participation. For group case studies, they purposively selected three adolescent groups formed at the start of the intervention and documented their engagement over the intervention period using open-ended notes and observation. Two adolescent groups were in clusters situated farthest from the main market town, and the third was in a multi-lingual community. Finally, the process evaluation officer interviewed external stakeholders in health and education who had engaged with intervention activities, including secondary and high school teachers (n = 8), anganwadi (Integrated Child Development Services) workers (n = 2), auxiliary nurse midwives (n = 1) and accredited social and health activists (n = 2). Audio recordings of interviews and discussions were transcribed using gisted transcription and translated into English during transcription or from completed Hindi transcripts. (See Supplementary File S4 and Table S4 for the process evaluation plan).

### 2.5 Intervention

**Fig. 2:**
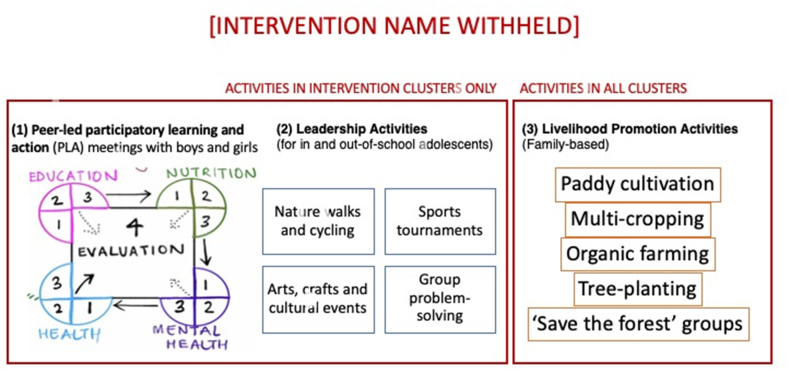
Overview of the intervention.

The intervention had three components: (1) participatory adolescent groups, (2) youth leadership activities, and (3) livelihood promotion (Fig. 2). Participatory adolescent groups were the main component . Adolescents could attend all or as many components as they liked, with no required minimum frequency of participation. All components were developed and supported by the implementation partner, with input from local stakeholders (teachers, parents, adolescents, and representatives of local health or social care organisations) during consultation workshops. The intervention was delivered by a community youth team comprising three types of staff: (1) peer facilitators called *yuva saathi*, (2) youth leadership facilitators, and (3) livelihood promoters. The community youth team received periodic residential training in delivering the intervention using participatory approaches, communication skills, and working with adolescents and families. Training was supplemented with regular supervision, and up to 20% of participatory adolescent groups were observed by coordinators and supervisors. English and Hindi versions of intervention manuals and facilitation guides for the community youth team are available to download with this article (doi:10.5522/04/21534990) .

#### 2.5.1 Participatory adolescent groups

Participatory adolescent group activities drew on previous research on participatory learning and action and on RKSK's priority areas (Nair et al., 2017, Tripathy et al., 2010).Female and male peer facilitators called *yuva saathi* (“friend of youth”) and aged 20–25 years facilitated monthly participatory group meetings with adolescent girls and boys in a local community space over a period of 33 months. During the first five meetings, groups discussed local influences on adolescents' health, strategies to include the most vulnerable adolescents in meetings, as well as adolescents' own needs and expectations. Parents, teachers, and frontline health workers were encouraged to participate. *Yuva Saathi* and adolescent groups then worked through four consecutive ‘mini’ participatory learning and action (PLA) cycles on education, nutrition, health and mental health and violence. Gender equity was not covered as a separate PLA cycle, and instead was treated as a cross-cutting issue that featured in adolescents' experiences of education (e.g. girls having less opportunity than boys to complete secondary schooling), nutrition (e.g. inequitable intrahousehold allocation of food with girls receiving less nutritious food), health (e.g., sex-specific pubertal changes), and mental health and violence (e.g. girls experiencing street harassment). Each mini-PLA cycle had six meetings focused on: (1) identifying and prioritising problems relating to the focal area (e.g. education); (2) understanding causes and finding solutions for two or three prioritised problems in the focal area (e.g. discontinued education, lack of access to school-related entitlements); (3) planning strategies and sharing out responsibilities; (4) implementing chosen strategies and learning about existing entitlements; (5) implementing chosen strategies and discussing alternative ones; (6) planning for a community meeting. Implementation of chosen strategies continued till the end of the intervention. Each mini-PLA cycle was followed by a wider community meeting in which parents, teachers and frontline workers were invited to hear about the adolescents' prioritised problems and support their strategies. Further information on each phase is included in Supplementary File S5 and Table S5.

We expected PLA cycles to improve adolescents' knowledge about health, education, nutrition and gender equity as well as related local public services and entitlements and increase their confidence in sharing their needs with peers and parents, and in addressing issues related to their own health and wellbeing. We also expected PLA cycles to increase parents', teachers' and other community members’ interest in supporting adolescent groups and their strategies.

#### 2.5.2 Youth leadership activities

The second intervention component was a programme of youth leadership activities. These took place every two months and were promoted by the *yuva saathis* during PLA meetings. They were designed by local youth leadership facilitators who were recruited based on prior experience, and activities were intended to be fun, build adolescents’ confidence, and keep them engaged in PLA meetings. Activities included nature walks, sports tournaments, cycling and group problem-solving conducted in the village, in local sports facilities or playgrounds, or in the surrounding forests and woodland.

#### 2.5.3 Livelihood promotion

Our formative research found that adolescents and their parents valued practical, livelihoods-related training in farming and environmental management. The final intervention component, livelihood promotion, was offered to both intervention and control groups as a common benefit, while the first two components were offered only in intervention clusters. Livelihood promoters met with adolescents and their parents every three months in local community spaces, fields, or close to a local market for season-focused activities including practical demonstrations of paddy cultivation, multi-cropping, organic farming techniques, tree planting, kitchen gardening and to revive ‘save the forest’ groups (*van samitiy*). This third component aimed to provide adolescents with practical skills to improve their families' food security and income.

#### 2.5.4 Supervision and cost

Community youth team members met monthly to coordinate their activities. A total of 30 *yuva saathi* paid INR 5000 (c. USD 60) per month were supervised by four coordinators and two supervisors who met with them fortnightly and provided support in the field. In addition, there were four youth leadership facilitators and two livelihood promoters paid INR 15000 (c. USD 180) monthly each. A budget of INR 275,596 (USD 3,329) was allocated for training, INR 223,637 (USD 2,701) for review meetings and workshops, and INR 191,638 (USD 2,315) for agricultural and sports activities. The average annual cost of running all three components was INR 4,966,270 (USD 59,993).

### 2.6 Outcomes

The trial's three primary outcomes were: (1) percentage of adolescent girls currently attending school or college; (2) mean dietary diversity score (based on 24-h recall) (FAO and FHI 360, 2016); and (3) mean score on the Brief Problem Monitor-Youth (Achenbach et al., 2011, p. 33). Twelve secondary outcomes captured indicators on girls' empowerment and decision-making, emotional and physical violence, resilience (Jefferies et al., 2019) and self-efficacy (Schwarzer et al., 1995), school attendance, and uptake of school-related entitlements. Eight exploratory outcomes assessed nutritional and anthropometric indicators, menstrual hygiene, knowledge of sexual and reproductive health. All outcomes were pre-specified.

### 2.7 Statistical methods

The number of clusters in the trial was based on the size of the study area, which was determined by logistical and financial constraints. We estimated that 4400 adolescent girls aged 10–19 lived in study sites, with c.115 in each cluster, based on a crude birth rate of 23 per 1000 population in West Singhbhum district. We revised our estimates after the baseline survey where we were able to interview 3324 (82%) of an estimated 4068 adolescent girls, which yielded a mean cluster size of 87 (SD 29·9), a coefficient of variation of cluster sizes of 0·3 and provided baseline scores and intracluster correlation (ICC) values for primary outcomes. The trial had 80% power to detect an increase in the proportion of girls attending school or college (ICC, 0·03) from 69% to 78%, an increase in the mean dietary diversity score (ICC, 0·4) from 3·4 (SD, 1·4) to 4·3, and a decrease in Brief Problem Monitor-Youth score (ICC, 0·39) from 6·0 (SD, 4·3) to 3·4, at 5% significance level. We aimed to interview all eligible residents and hence roughly the same number of adolescent girls in baseline and endline surveys.

All primary and secondary analyses were by intention to treat, following an analysis plan approved by the trial Data Monitoring Committee in December 2019 and revised in July 2020. We used mixed-effects models to adjust for clustering. We assessed the effect of the intervention in linear or logistic regression as appropriate, baseline and endline data were analysed together but cross-sectionally, in that individual participant data were not matched in the two surveys. Outcomes were converted to binary, continuous or categorical variables following strategies suggested by the developers of each tool. Where endline measurements relied on updated versions of tools used at baseline and thus had different total scores (for example, the Brief Problem Monitor – Youth for mental health used at endline included an additional subscale which was not in the baseline Brief Problem Checklist), we converted baseline and endline values to proportional scores to aid interpretation of analyses. Following the constrained baseline approach (Copas and Hooper, 2019; Hooper et al., 2018) we included in our regression models an indicator of time (baseline and endline) and an indicator of intervention, coded one for adolescents measured at endline in intervention clusters and zero otherwise. To reach a conclusion about whether an overall benefit of the intervention had been demonstrated or not, based on the three primary outcomes, we prespecified ‘overall trial success’ in the protocol to include a significant (two-tailed p < 0·05) benefit for at least one outcome, in conjunction with a collectively ‘positive signal’ for the other two outcomes. We defined a ‘positive signal’ as at least one of the two outcomes in the direction of benefit, neither outcome showing significant harm, and if for one outcome the direction of effect was towards benefit and for the other it was towards harm then the (two-tailed test) p-value for the former outcome would need to be smaller than that for the latter.

For each primary and secondary outcome, we adjusted for asset quintile, tribal status, and age, and for strata, which were specified a priori. Post-priori, we included occupation of the main wage earner as an additional factor due to small imbalances in proportions between intervention and control groups observed at endline. Unadjusted models included baseline data on outcomes where available. Data on exploratory outcomes were analysed using descriptive statistics to compare intervention and control groups.

We conducted three sub-group analyses for primary outcomes using the same analytic approach as the main analyses, to assess differential intervention effects by age group (10–14 years and 15–19 years), wealth quintile (lowest two quintiles and upper three quintiles), and exposure to the intervention (attendance at over 50% of group meetings and leadership activities, and less than 50% attendance at group meetings and leadership activities).

Quantitative process evaluation data on composition of adolescent groups, socio-demographic characteristics of adolescents at each meeting and attendance records were analysed using descriptive measures (mean, median, rank, frequency) aggregated at the cluster level or by time (calendar month or meeting number). Where available, data were stratified by adolescent gender, age group and school enrolment status. We analysed quantitative process data using MS Excel and Stata version 16.

For qualitative data, we primarily used the framework method approach with a coding framework developed using the intervention's theory of change (Gale et al., 2013). Multi-level codes were defined by intervention resources, community activities, and anticipated early and later effects on adolescent and caregivers' learning, motivation and action on health outcomes. We conducted additional inductive thematic analysis to identify emergent themes related to intervention context and mechanisms not covered in the theory of change. We attempted to triangulate findings from different data sources to examine concordance between qualitative quotes and quantitative tables describing similar processes. We used NVivo version 12 and MS Excel to analyse qualitative data.

The trial was suspended on the 14^th^ of March 2020 due to the emergence of COVID-19 in India, in advance of the first phase of national lockdown which started on the 24^th^ of March 2020. Our decision was guided by concerns about community transmission of SARS-CoV-2 during group intervention activities as well as in-person, close-contact data collection for the endline survey and process evaluation. In June 2020, following consultation with the intervention team, trial funder and Data Monitoring Committee, the trial was stopped because school closures, psychological stress, and food insecurity related to the effects of lockdown and the pandemic would have affected all three primary outcomes. Additional data collected (in-person or using remote methods) at that point would not have yielded valid results on education, nutrition, or mental health. We also re-calculated detectable differences for primary outcomes and estimated that the amount of survey data already collected would be sufficient to complete primary analyses with reasonable adjustments to our analysis plan and some loss of statistical power. We deviated from our pre-specified statistical analysis plan to use generalised estimation equations to estimate effects on primary outcomes, opting for mixed-effects models to better accommodate a larger than expected proportion of missing data at endline due to early stopping (see Supplementary File, S6 for a full explanation). KB and the trial statistician were masked to treatment during statistical analyses of primary outcomes. We used Stata version 16 for statistical analyses.

The trial is registered as ISRCTN17206016.

## 3 Results

### 3.1 Trial profile and follow-up

**Fig. 3:**
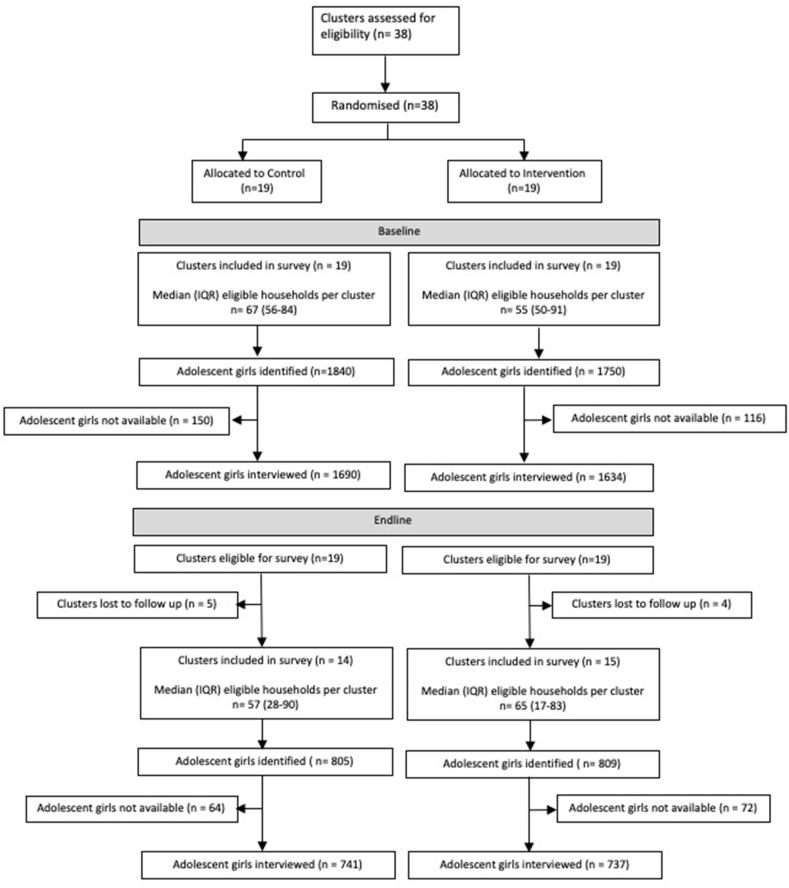
Trial profile.

Fig. 3 is the trial profile. The baseline survey was conducted between June 2016 and January 2017, and the endline survey between February and March 2020. We interviewed 3324 (82%) of an estimated 4068 eligible adolescent girls aged 10–19 years in 38 study clusters in the baseline survey (1690 (51%) from clusters subsequently allocated to the control group, and 1634 (49%) to the intervention group). Nine clusters (four intervention and five control) were lost to follow up when endline survey data collection was suspended before the anticipated end date of May 2020 due to the COVID-19 pandemic. We interviewed 1478 adolescents (737 in intervention clusters, 741 in control clusters) in 29 clusters (15 intervention, 14 control), reaching 44% of our target sample. Our combined baseline and endline analysis dataset was 72% complete (4802 of 6648 target interviews completed). As a result, detectable differences in outcomes were larger than estimated under the scenario of complete follow-up at both time points (Supplementary File, S6). The early stopping of our endline survey might have introduced selection bias. However, the baseline distribution of socio-economic characteristics in clusters that were successfully followed up was similar to that in the full sample, indicating that our reduced sample in the endline survey was not remarkably different to the overall population of adolescents in the study (Supplementary File S7, Table S7). In addition, adolescent girls in intervention and control groups had similar socioeconomic and demographic characteristics at endline (Table 1). (See Supplementary File S8, Table S8 for baseline data). Over half of adolescents in both arms aged 10–14 years, fewer than one in five owned a personal mobile phone, and one in ten were married. In both arms, over 75% belonged to scheduled tribe communities and the most common source of household income was daily wage labour. Fewer than 10% had access to toilet facilities in the home.

**Table 1:** Characteristics of participants by study arm at endline.

Characteristic	Control	Intervention	All
Adolescent girls interviewed at baseline	741 (50%)	737 (50%)	1478
Household location			
Main village	576 (78%)	587 (80%)	1163 (79%)
Hamlet	165 (22%)	150 (20%)	315 (21%)
Class or caste status			
Scheduled tribe	624 (84%)	554 (75%)	1178 (78%)
Scheduled caste	8 (1%)	46 (6%)	54 (4%)
Other backward caste	107 (14%)	132 (18%)	239 (16%)
Other	2 (0·3%)	5 (0·7%)	7 (0·5%)
Tribe (n = 1178)			
N	624	554	1178
Ho	624 (100%)	529 (95%)	1153 (98%)
Santhal	0 (0%)	20 (4%)	20 (1.7%)
Munda	0 (0%)	2 (0·4%)	2 (0·2%)
Other	0 (0%)	3 (0·5%)	3 (0·2%)
Religion			
Sarna	609 (82%)	502 (68%)	1111 (75%)
Hindu	103 (14%)	141 (19%)	244 (17%)
Christian	24 (3%)	62 (8%)	86 (6%)
Other	5 (1%)	32 (4%)	37 (3%)
Adolescent's marital status			
Not married	707 (95%)	699 (95%)	1406 (95%)
Married	34 (5%)	38 (5%)	72 (5%)
Literacy			
Cannot read	172 (23%)	78 (11%)	250 (17%)
Reads with difficulty	257 (65%)	253 (34%)	510 (34%)
Reads easily	312 (42%)	406 (55%)	718 (49%)
Personal mobile phone ownership	115 (15%)	126 (17%)	241 (16%)
Household maintains small kitchen garden	196 (26%)	239 (32%)	435 (29%)
Multidimensional poverty quintile			
*Highest (least poor)*	223 (30%)	148 (20%)	371 (25%)
*Second highest*	129 (17%)	114 (15%)	243 (16%)
*Middle*	151 (20%)	150 (20%)	301 (20%)
*Second lowest*	118 (16%)	147 (20%)	265 (18%)
*Lowest (poorest)*	120 (16%)	178 (24%)	298 (20%)
Daily paid labour (MNREGA)	131 (18%)	185 (25%)	316 (21%)
Toilet			
Not improved	166 (22%)	164 (22%)	330 (22%)
Improved	211 (28%)	205 (29%)	416 (29%)
Field	364 (49%)	368 (50%)	732 (49%)
Age group			
10–14 years	419 (57%)	429 (58%)	848 (57%)
15–19 years	322 (44%)	308 (42%)	630 (43%)

Data are n (%). MNREGA = Mahatma Gandhi National Rural Employment Guarantee Act, a national programme to provide wage employment to households in rural areas.

### 3.2 The intervention in practice

The community youth team delivered 3694 participatory adolescent group meetings and conducted 18 leadership training activities in 19 intervention clusters between June 2017 and March 2020. They conducted 12 livelihood promotion activities across all 38 clusters. In the endline survey, 71% (524 of 737) of adolescent girls in intervention clusters recalled ever attending a participatory group meeting since the start of the intervention, of whom 88% (407 of 524) had attended in the three months before the survey. In the 12 months preceding the survey, 48% (356 of 737) had participated in leadership activities. Corresponding data for control clusters were 24% (175 of 741) and 48% (83 of 175) for participatory group meetings and 20% (147 of 741) for leadership activities. Since adolescents were asked if they had attended these activities in their village or hamlet and contamination would have required the community youth team to go to intervention areas to conduct these, the data point to similar initiatives by other organisations, but we do not have data to verify this. Participation in livelihood promotion activities in the year preceding the survey was 12% in control clusters and 22% in intervention clusters.

Data from registers showed that between 91 and 109 (mean 104, SD 3·5) participatory adolescent groups met every month across the 19 intervention clusters. The mean number of participants per month across all groups was 2182 (SD 372), of whom 80% were aged 10–19. Other attendees included interested adults and children in the community (9%), parents of adolescent group members (8%), young adults aged 20–24 (2%), and health workers and teachers (1%). Overall, 57% of adolescents in meetings were female, 66% were aged 10–14, 88% were in formal education, and 2% were married. The 1187 (32%) meetings that took place between October 2017 and May 2018 were conducted in sex-segregated groups (697 meetings for 104 girls' groups and 490 meetings for 64 boys' groups) at adolescents’ request to discuss sex-specific biological changes and social problems faced by young people.

During the intervention, 4610 adolescents formally joined their local participatory adolescent group, though the duration of membership and attendance varied substantially. The number and size of groups was determined by the number of adolescents living in the village or hamlet, and their popularity among adolescents. The mean number of registered participants in a study cluster in any six-month period was 146 (SD, 32). The median number of meetings attended was nine out of 30 (interquartile range (IQR), 4, 16), with higher exposure among girls (median, 10, IQR, 5, 18) than boys (median 7, IQR, 3, 13).

The three most common health problems prioritised by adolescent groups were early marriage, substance use and poor (menstrual) hygiene. For mental health and violence, adolescents prioritised physical violence at home, emotional violence at home, street harassment (experienced by girls) and bullying (experienced by boys). For nutrition, adolescents prioritised unsafe food handling, inadequate dietary diversity, and unkempt kitchen gardens. The three most common education problems prioritised by adolescents were discontinued education, gender discrimination in favour of boys' education, and lack of access to school-related entitlements. Strategies to address these problems often involved supported referrals by the community youth team to education or health services, individual or family-based counselling provided by a community health worker or experienced intervention staff, mobilising community support for adolescents’ activities, and involving marginalised adolescents in activities to make groups more inclusive.

### 3.3 Results for primary outcomes

Mean dietary diversity score was 4·0 (SD 1·5) in intervention clusters and 3·6 (SD 1.2) in control clusters, with an ICC of 0·25 (95%CI 0·13, 0·38). Mean Brief Problem Monitor-Youth (mental health) score was 12·5 (SD 6·0) in intervention clusters and 11·9 (SD 5·9) in control clusters, with an ICC of 0·34 (95%CI 0·19, 0·48). The proportion of adolescent girls currently enrolled in school was 70% in intervention clusters and 63% in control clusters, with an ICC of 0·05 (95%CI 0·01, 0·08). We found no intervention effects on dietary diversity score (aD 0·34; 95%CI -0·23, 0·93, p = 0·242), proportional mental health score (aD 0·02, 95%CI -0·06, 0·13, p = 0·610) or the proportion of girls attending school (aOR 1·39, 95%CI 0·89, 2·16, p = 0·142) (Table 2).

**Table 2:** Adjusted and unadjusted effects of intervention on primary and secondary outcomes.

	Control	Intervention	Adjusted effect (95% CI) †	p value	Unadjusted effect (95% CI) ‡	p value
**Primary outcome**						
N	741	737				
Adolescent girls attending school or college	471 (64%)	518 (70%)	adjOR 1·39 (0·89, 2·16)	0·142	OR 1·23 (0·88, 1·72)	0·216
N	741	737				
Dietary diversity score (24-h recall)	3·6 (1·5)	4·0 (1·3)	adjDiff 0·34 (−0·23, 0·92)	0·242	Diff 0·34 (−0·27, 0·95)	0·272
N	741	737				
Mental health score, based on the Brief Problem Monitor-Youth*	11·9 (5·9)	12·5 (6·0)	adjDiff 0·02 (−0·06, 0·12)	0·610	Diff 0·03 (−0·06, 0·13)	0·562
**Secondary outcome**						
N	741	736				
Girls making decisions independently and with others about the food they eat including how much they eat and what types of food they eat	211 (29%)	295 (40%)	adjOR 2·10 (0·75, 5·91)	0·159	OR 1·88 (0·64, 5·49)	0·248
N	741	737				
Gender role attitudes	4·0 (1·4)	4·3 (1·4)	adjDiff 0·04 (−0·48, 0·56)	0·884	Diff 0·03 (−0·51, 0·56)	0·927
N	741	736				
Girls making decisions independently and with others about						
Choosing friends	148 (20%)	267 (36%)	adjOR 1·41 (0·45, 4·47)	0·552	OR 1·19 (0·37, 3·82)	0·766
How and when to spend money	263 (34%)	322 (44%)	adjOR 1·53 (0·59, 3·96)	0·380	OR 1·33 (0·49, 3·59)	0·572
What purchases to make	273 (37%)	327 (44%)	adjOR 1·6 (0·58, 4·39)	0·360	OR 1·48 (0·56, 3·90)	0·430
N	741	737				
Self-efficacy score on the Schwarzer General Self-Efficacy Scale§	11·7 (3·1)	12·5 (2·8)	adjDiff 0·36 (−0·76, 1·48)	0·529	Diff 0·41 (−0·73, 1·56)	0·478
N	741	737				
Mean score on the Child and Youth Resilience Measure 11-item version§	23·9 (4·2)	25·2 (3·6)	adjDiff 0·16 (−1·43, 1·76)	0·843	Diff 0·41 (−1·22, 2·04)	0·622
N	741	737				
Girls report experiencing emotional violence in the past 12 months	608 (82%)	649 (88%)	adjOR 2·22 (0·66, 7·44)	0·198	OR 2·06 (0·62, 6·82)	0·236
N	741	737				
Girls report experiencing physical violence in the past 12 months	394 (53%)	439 (60%)	adjOR 1·91 (0·66, 5·54)	0·234	OR 1·79 (0·69, 4·63)	0·227
N	741	737				
Girls report intervening to reduce emotional violence against their peers in the past 12 months §	420 (57%)	427 (58%)	adjOR 1·04 (0·40, 2·67)	0·943	OR 1·19 (0·45, 3·18)	0·720
N	741	736				
Girls report intervening to reduce physical violence against their peers in the past 12 months §	291 (61%)	317 (43%)	adjOR 1·39 (0·45, 4·35)	0·567	OR 1·54 (0·49, 4·79)	0·456
N	471	518				
Girls report being absent from school in the past two weeks	322 (68%)	310 (60%)	adjOR 0·53 (0·19, 1·47)	0·220	OR 0·58 (0·22, 1·55)	0·277
N	471	518				
Girls accessing at least one school-related entitlement (cash, bicycles, books, or midday meal scheme) §	413 (88%)	479 (92%)	adjOR 2·01 (1·11, 3·64)	0·020	OR 1·78 (1·02, 3·11)	0·041
N	168	148				
Girls report drinking alcohol in the past month	140 (83%)	100 (68%)	adjOR 0·24 (0·05, 1·27)	0·093	OR 0·19 (0·04, 0·91)	0·038

Data are n (%) or mean (SD). Effect estimates are differences (Diff) or odds ratios (OR). *BPM-Y scores out of a maximum of 40. Raw scores normalized to compare with baseline data collected using an earlier version of the tool (Brief Problem Checklist) with a maximum score of 24. The BPM-Y has a six-item Attention Problems scale in addition to Internalising, Externalising and Total Problems scales. Data were log-transformed for regression modelling. † Adjusted for age, strata, asset quintile, livelihood, and tribal status. ‡ Unadjusted estimates include baseline values § Not measured at baseline * † ‡ § ¶ ‖

### 3.4 Results for secondary and exploratory outcomes

We found no effect of the intervention on the proportion of adolescent girls who made decisions independently and with others about the food they ate, their friends, how they spent their money and what items they purchased (Table 2). Computed scores for resilience and self-efficacy were slightly higher in intervention clusters, but there were no differences between arms in unadjusted or adjusted regression models (Table 2). We did not detect any difference in girls’ experiences of emotional or physical violence, reports of intervening to reduce violence against their peers in the past year, or consumption of alcohol in the past month (Table 2), though the crude scores for these outcomes were slightly higher among participants in the intervention group. While we did not detect effects on school absenteeism in the two weeks preceding the survey (Table 2), girls in intervention clusters were two-times more likely to access at least one school-related entitlement (aOR 2·01; 95%CI 1·11, 3·64, p = 0·020).

Results on exploratory outcomes related to nutrition indicate that uptake of food and nutrition-related entitlements was low across groups and over time, and the prevalence of anthropometric status indicative of undernutrition remained high (Table 3). The proportions of adolescent girls who knew that abortion is legal and reported using sanitary napkins or clean cloths during their period was higher in both arms after the intervention, while fewer adolescents were able to describe correct ways of using contraception methods in the endline survey compared to frequencies for each group before the intervention (Table 3).

**Table 3:** Exploratory outcomes by study arm.

Exploratory outcome	Control		Intervention	
	Baseline	Endline	Baseline	Endline
**Outcomes related to nutrition**				
n	399	257	396	320
Girls report taking at least four iron and folic acid supplements in the past month	108 (27%)	76 (30%)	138 (35%)	104 (33%)
n	1690	741	1634	737
Girls report receiving take-home rations in the past month	64 (4%)	8 (1%)	35 (2%)	22 (3%)
n	1649	721	1624	716
Underweight (<-2SD median BMI for age and sex)	177 (11%)	86 (12%)	173 (11%)	85 (12%)
n	1689	721	1633	723
Stunted (<-2SD median height for age and sex)	779 (46%)	284 (40%)	714 (44%)	314 (43%)
n	1690	734	1634	733
Mid-upper arm circumference (cm)	21.4 (2.8)	21.3 (2.9)	21.3 (2.8)	21.3 (2.9)
**Outcomes related to sexual and reproductive health**				
n				
Girls aged 15–19 and all married girls have correct knowledge about contraception				
n	377	120	298	169
Contraceptive pill	91 (24%)	22 (18%)	71 (24%)	19 (11%)
n	207	77	156	140
Condoms	83 (40%)	15 (19%)	79 (51%)	33 (24%)
n	90	48	92	79
Intra-uterine device	25 (28%)	38 (79%)	44 (48%)	38 (48%)
n	1131	491	1089	512
Girls use sanitary napkins or clean cloths during their period	841 (74%)	475 (97%)	845 (78%)	469 (92%)
n	823	322	724	310
Girls aged 15–19 and all married girls know that abortion is legal	55 (7%)	96 (30%)	49 (7%)	85 (27%)

Data are n (%) or mean (SD). BMI = body mass index.

Pre-specified sub-group analyses examining effects by adolescent age and wealth quintile showed little evidence of intervention effect being modified for all three primary outcomes, though the regression models on current educational status by wealth quintile sub-groups failed to converge, likely due to sparse data. We were unable to examine sub-group effects by intensity of exposure to the intervention due to sparse data (Table 4).

**Table 4:** Intervention effects on primary outcomes by age group and wealth quintile.

	Adolescent girls attending school or college				Dietary diversity score, based on 24-h recall				Mental health score, based on the Brief Problem Monitor-Youth^a^			
	Control	Intervention	Adjusted effect (95% CI) †	p value	Control	Intervention	Adjusted effect (95% CI) b	p value	Control	Intervention	Adjusted effect (95% CI) †	p value
**Age group**												
n	419	429			419	429			419	429		
10–14 years	338 (81%)	367 (86%)	1·36 (0·79, 2·33)	0·259	3·6 (1·2)	4·0 (1·4)	0·45 (−0·16, 1·07)	0·149	12·0 (6·2)	11·9 (5·9)	0·03 (−0·6, 0·14)	0·509
n	322	308			322	308			322	308		
15–19 years	133 (41%)	151 (49%)	1·27 (0·75, 2·17)	0·367	3·6 (1·3)	4·0 (1·6)	0·12 (−0·44, 0·69)	0·662	11·8 (5·5)	13·2 (6·1)	0·02 (−0·7, 0·12)	0·654
**Wealth quintile**^c^												
n	389	475			389	475			389	475		
Least poor quintiles	284 (73%)	353 (74%)			3·7 (1·3)	4·1 (1·5)	0·31 (−0·33, 0·94)	0·343	11·3 (5·5)	12·3 (6·2)	0·02 (−0·07, 0·13)	0·582
n					352	262			352	262		
Poorest two quintiles	187 (53%)	166 (63%)	1·64 (0·84, 3·20)	0·146	3·4 (1·1)	3·9 (1·4)	0·23 (−0·28, 0·75)	0·371	12·6 (6·3)	12·9 (5·8)	0·05 (−0·05, 0·17)	0·347

Data are n (%) or mean (SD). Effect estimates are regression beta-coefficients or odds ratios.

a BPM-Y scores out of a maximum of 40. Raw scores normalized to compare with baseline data collected using an earlier version of the tool (Brief Problem Checklist) with a maximum score of 24. The BPM-Y has a six-item Attention Problems scale in addition to Internalising, Externalising and Total Problems scales. Data were log-transformed for regression modelling. Coefficients are proportional scores.

b Adjusted for age, strata, asset quintile, livelihood, and tribal status.

c Adjusted regression model for primary outcome on education for least poor quintiles did not converge.

### 3.5 Qualitative findings

Qualitative process data on group composition and contextual factors affecting the intervention lend further insight. Older adolescents, boys, and out-of-school adolescents attended group activities less frequently because they contributed to or supported household economic activity, or spent their spare time studying for exams. Some also felt embarrassed to come to meetings because they had dropped out of school and could not read or write. *“I don't attend meetings regularly because I have housework, coaching classes and self-study which takes up a lot of time. My board exams are also approaching, so I have to prepare for that. My mother is an agricultural worker and often she is not at home, so it's hard to come to meetings”* – Adolescent girl, 17 years old

*“I am 19 years old and study in the 9*^*th*^*standard but I am not currently attending school, because I am married. I haven’t been attending school for the last month. It’s not that my family have stopped me going. I’ve decided not to go because I was thinking about who will give my wife money to buy oil and soap [consumables]. Yes, it’s possible for me to resume school after speaking to the head-teacher, but then who will give my wife money to buy oil and soap, that’s what I’m thinking … And women in the village will comment on the fact that I’m going to school even though I’m now married.”* - Adolescent boy, 19 years old

*“Adolescent boys who sit in the shop and gossip, go to village market, travel to other places for football tournament … Adolescent boys feel ashamed … those who cannot write their names … so they do not come to the meeting.”* – *Yuva saathi*

Younger adolescents, who had greater exposure to intervention activities, often asked for further clarification or simpler language during discussions, and sometimes struggled to participate in activities that involved decision making and collective action.

*“In the meeting, all adolescents participated but the adolescents aged 10–13 were finding it difficult to understand. It was a little difficult on the part of younger adolescents where there was a group activity related [to] help [or] seeking support. They could not decide where to move as the situation demanded.”* – Community Youth Team Supervisor

While quantitative data suggest that health workers' and schoolteachers’ engagement with group meetings was low, qualitative data suggest that these interactions were largely positive and involved collaborative or supportive actions initiated by the community youth team, who were able to engage with adolescents as peers on issues that health workers and teachers could not effectively address.

*“Certainly, re-enrolments have happened. Earlier, once they became irregular in coming to school, they wouldn’t come back to school again for years and years. Now after re-enrolment in classes the tendency of school irregularity has reduced. Beside the re-enrolment they [yuva saathi] also conduct all these competition and sports activities, so that the children develop interest for education.”* – Schoolteacher 1

*“As I said earlier, they [adolescents] cannot share their problem with us [older adults] openly. Therefore, it is the yuva saathi with whom they are comfortable in sharing any issues. Any issue related to SRH [sexual and reproductive health] and about monthly periods, they are easily sharing with yuvasathi.”* – Accredited Social Health Activist

The community youth team served as liaison agents for education, helping adolescents and parents to navigate school bureaucracy, re-enrolments, and entitlements. Our data suggest that this mechanism was not replicated for mental health or nutrition, possibly due to limited availability and implementation challenges associated with food welfare and mental health services at the community level, and adolescent girls’ difficulty in negotiating intrahousehold food allocation at the family level. Adolescents who reported increased knowledge of nutrition were likely from more food secure families, and those who felt that participating in leadership activities and spending time with friends had improved their lives were unlikely to have substantial underlying stressors.

*“From this meeting we learnt which food came under which category and we had come with vegetables, dal, chawal [rice] from each of our houses … foods that we like. We learnt that green leafy vegetables help in making blood and we learnt which foods are rich in protein and which foods are good for health.”* – Adolescent girl’s feedback after a meeting on nutrition

*“I have been to most PLA meetings. If I can’t attend, I ask my friends to share what happened. I have been going for over two years. I remember going to meetings about who adolescents are, and I went trekking in the forest. I think it has improved my life, I feel I have changed. Earlier, there were no meetings, but now we have yuva saathi who can teach us and discuss things. I like the knowledge about school-based entitlements (school bag, bicycle, stipend). If I don’t like something, I say, or if I don’t understand, they explain it again. I think meetings should continue.”* – Adolescent boy, 14 years old

Changes in parents' learning are likely, but their motivation or actions to improve adolescent wellbeing because of the intervention were less clear. In instances where parents inflicted emotional violence on adolescents (for example, preventing them from attending school to look after younger siblings or work) or when household dysfunction contributed to adolescents’ mental distress (e.g., addiction or domestic violence), the intervention did not sufficiently engage with and engender sustained support from parents.

*“Parents said that those things we have never seen earlier, [but now we] can understand the issue seeing the picture card. We will not fight in front of our children. We will not scold them. Because there is [a] chance that they will run away and may attempt suicide*.” – Community youth team supervisor reporting parents' feedback

*“I think the intervention could be improved by discussing strategies to address parent-adolescent conflict and disagreements.”* – Adolescent boy, 17 years old

The most salient contextual factor that impeded larger impact is household poverty, which not only reduced the resources and support available to adolescents to improve their health, but also demonstrates how interconnected the study's primary outcomes are for the poorest adolescents in this rural population.

*“The one and only reason [for limited engagement with the intervention] is their poverty. In poor families in our village, children are engaged in grazing goats and bullocks, and during agricultural seasons are engaged in farm related work, and [a] few are engaged in their household chores, all these are because of their poverty. Those who work in town or nearby cities are also not so much engaged.”* – School headmaster

*“First, I would like to say their parents are the barriers. Because in parents' meeting, they say “we are poor, we are farmers, if we don’t send our children for labour, how will we feed them?” They only share their issues. We can’t solve their issues. We can’t feed them. We can’t pay their fees, and we can’t give them labour … whatever they get. What we can do, we can take admission [to school] only. But in a year, 70% attendance is a minimum requirement. They don’t fulfil that … And so they are deprived of writing [end of year] exams.”* – School teacher 2

One qualitative data gap is that data collected by the community youth team had fewer instances of detailed feedback on how the intervention could be improved or more focused on achieving equity, or why it may not have affected adolescent mental health and nutrition. Data were skewed towards individual success stories and positive feedback on the intervention's impact on education. Due to early stopping, other study investigators were unable to collect more independent data from participants to gain a deeper understanding of specific barriers to impact on mental health and nutrition.

No adverse events or harms attributable to the intervention were reported.

## 4 Discussion

Our study found that an intervention comprising participatory adolescent groups, youth leadership training and livelihood promotion activities delivered by a community youth team did not significantly improve adolescent girls' mental health, dietary diversity, or school attendance in rural eastern India. While the intervention did not meet pre-specified criteria for success, estimated effects did not indicate any risk of harm, and confidence intervals did not exclude important intervention benefits. The findings suggest the intervention may have increased uptake of education-related entitlements, though this was the only significant finding across 12 secondary outcomes tested. Process evaluation findings indicated that adolescents aged 10–14 had greater engagement with the intervention than adolescents aged 15–19, adolescent boys made up 40% of participatory adolescent groups, and that household poverty was a substantial barrier to enlisting the resources and support necessary to see positive changes to the trial's primary outcomes.

Our findings corroborate the small evidence base on peer-facilitated interventions in India and provide further insight. PAnKH (Promoting Adolescent eNgagement, Knowledge and Health), a three-arm cluster RCT in the state of Rajasthan tested a gender transformative curriculum delivered by peer-educators, comparing a girls-only model to an integrated model which included community members as well as adolescent boys (Institute for Fiscal Studies, 2018). Compared to a control group receiving standard government services, the gender-transformative intervention had positive effects on delayed marriage and school retention in the girls-only model, with very small improvements in girls’ mental health only in the arm that included a strong community engagement component and meetings with adolescent boys aged 15–18. As in our study, the benefit of peer-facilitation for education-related outcomes in PAnKH is clearer than that for mental health. However, the PAnKH trial was not published as a peer-reviewed article.

A cluster trial to promote gender egalitarian norms among boys in rural areas of the state of Bihar, testing the Do Kadam (*Two Steps*) programme delivered by peer mentors and combined with sport coaching, found greater benefits for younger boys (13–14 years) than older boys (15–19 years) (Gupta & Santhya, 2020). The sustained engagement of younger adolescent boys in the intervention suggests that there may have been impacts on boys' gender attitudes as a result of their participation which remain unobserved, but qualitative data describing opposing views from girls and boys in relation to boys’ behaviours preclude any decisive insight without further research.

Non-randomised studies in Rajasthan have found mixed effects of peer-facilitated curricula on knowledge, attitudes and behaviours related to menstruation among girls (Dwivedi et al., 2020), and gender, sexuality and violence among young men (Freudberg et al., 2018), but these were small studies with fewer than 200 participants, demonstrating feasibility more than effectiveness. A multi-site post-test survey following an intervention comprising peer-education and youth information centres in the states of Uttar Pradesh and Bihar found no effects of peer education, but some positive effects of youth information centres on reducing school dropout, early marriage and pregnancy (Mehra et al., 2018). However, the lack of control groups and randomisation limits comparison. Evidence from a mental health-focused pilot intervention in Dehradun, Uttarakhand using realist evaluation methods (Mathias et al., 2019) and a quasi-experimental approach (Mathias et al., 2018) offers insight into dose-response relationships and mechanisms between peer-education and mental health. For out-of-school adolescent girls and young women aged 12–24, a peer-facilitated mental health and resilience intervention improved anxiety and depression scores through 15 weekly sessions following a mental health curriculum called *Nae Disha*, of which the 106 participants attended an average of 13·8 (Mathias et al., 2018). A realist evaluation of the *Nae Disha* curriculum delivered to mixed-sex groups of 142 adolescents living with psychosocial disability identified the importance of increasing peer networks through new friendships, engendering parental support and providing skilled peer-facilitation for adolescents’ sense of social inclusion in their communities (Mathias et al., 2019). This evidence suggests the need for more frequent engagement than we offered, more targeted inclusion of out-of-school adolescents than we were able to achieve, and higher participation than we were able to sustain. While smaller, focused studies using non-randomised designs hint at improvements in mental health that can be sustained (Mathias et al., 2018, Mathias et al., 2019), effect sizes reported in randomised studies are modest (Leventhal et al., 2015). While anxiety and depression seem difficult to shift, The Girls First Resilience Curriculum implemented in schools in Bihar showed stronger improvements in primary outcomes on self-efficacy, emotional resilience and socio-emotional assets following an intervention with a strong leadership component (Leventhal et al., 2015). Improvements in psychosocial assets may be more attainable than psychosocial wellbeing through leadership training, suggesting that these elements of the intervention could have been intensified and delivered more frequently.

We did not find any effects on girls' dietary diversity, decision making around food, or nutritional status, and only 2% of adolescents reported access to food-related entitlements. This suggests that any supply-side issues in the provision of subsidised staple foods for poor households would not have been counterbalanced by our intervention's attempt to increase demand through a focus on nutrition education, kitchen gardening and promoting consumption of micronutrient-rich but energy-poor vegetables and fruits. Given the strong relationship between food access and nutritional status of adolescents in rural communities in India (Ganpule-Rao et al., 2020), this possibility is more worrying than surprising in a community with high levels of adolescent undernutrition and stunting. Against a backdrop of food insecurity and non-universal coverage and delivery of food subsidies for the poorest families and supplemental nutrition for undernourished adolescents, even the most effective participatory adolescent group intervention would struggle to improve the overall diversity of its members' diets. The contextual relationship between demand and supply of food-related entitlements requires greater investigation in this population.

Our study has four key limitations. First, we were unable to complete endline surveys as planned, and the trial had incomplete follow-up due to the COVID-19 lockdown which resulted in a substantial amount of missing data. While we were able to analyse data on primary endpoints successfully, our sub-group analyses were largely inconclusive, potentially due to loss of power, limiting insight on age- and wealth-based equity impacts of a peer-education model in this rural community. Second, like most trials of complex interventions, we included many trial outcomes, and whilst we pre-specified a strategy to handle the three primary outcomes, results for each secondary outcome should be treated with some caution due to multiple testing. Third, we relied on self-reported data for primary and secondary outcomes and were not able to verify responses with independent observation or cross-checking (for example, examining school registers to validate school enrolment data). It is possible that some outcomes were under-reported due to recall bias, or that questionnaires were not able to measure some outcomes with the same degree of reliability as more objective methods of assessment. Fourth, our qualitative process data are prone to social desirability bias since a large proportion of this data were collected by members of the intervention team rather than investigators external to the project. A more independent team engaged in data collection and iterative analysis throughout the intervention period would likely have given us a deeper understanding of barriers to success, particularly in relation to equity and age-related issues around engagement with intervention activities, and in the domains of mental health, violence and nutrition.

Our study has three important strengths. First, it is one of very few cluster-randomised controlled trials to shed light on the potential of community-based interventions to improve adolescent health and wellbeing in multiple domains, in alignment with national priorities and a wider strategy to use a peer educator model. Second, our trial was well-designed and included pre-specified methods published in a protocol with oversight from a data monitoring committee and prospective entry in a trial registry. Third, our study involved boys as participants in the intervention as well as the process evaluation, demonstrating that their participation in a group-based peer-led intervention can be sustained over a period of almost three years.

On balance, and viewed from the perspective of Nutbeam's model of levels of health literacy (Nutbeam, 2000), the intervention may have been sufficient to increase adolescents' functional health literacy but was not intensive enough to enable higher level interactive and critical health literacy. Through the intervention, adolescents were able to understand the determinants of health and obtain knowledge necessary to access help (functional health literacy), but not progress to achieve greater autonomy and power in individual decision-making and self-efficacy (interactive health literacy) or collectively act on wider social determinants of health (critical health literacy). It is possible that younger adolescents, who were more likely to sustain participation over time, were less able to build self-efficacy without the role-modelling of older adolescents and less equipped to create change without the support of parents and other adults. This is consistent with the conclusions of a systematic review (Siddiqui et al., 2020) as well as smaller qualitative studies conducted in rural India (Guha et al., 2019, Sharma et al., 2021) that peer education and participatory adolescent groups have been more effective at improving knowledge and attitudes than changing behaviours linked to health outcomes.

Our quantitative process evaluation data suggested that parents, health workers and teachers had limited engagement with participatory adolescent group meetings and other intervention components, which may have undermined our ability to improve outcomes in all three focal areas given their central role as guardians and gatekeepers.

Although we aimed to select outcomes potentially amenable to a community intervention using a theory of change built from extensive formative research, household poverty affected parents' ability to support adolescents’ strategies and limited the resources available to adolescents to improve their health.

Our findings are generalisable to similar rural, underserved areas of India with multiple adolescent health needs, and where a community-based adolescent health programme is underway or planned. Such initiatives need to adopt a more socio-ecological model of health promotion in which parents, teachers and frontline health workers are more actively engaged in adolescent health. The presence of other civil-society organisations (for e.g. Children in Need Institute (cini-india.org) and Centre for Catalysing Change (c3india.org) that work across eastern India) in Jharkhand is encouraging, and points to potential for further engagement with child and adolescent health in rural communities. Combining community-based interventions with initiatives to improve coverage and quality of health, education, nutrition, and social protection services would offer greater potential for peer-education and participatory approaches to succeed. Our study demonstrates that current RKSK strategies may need to be intensified and supported by access to high-quality mental health services in the community, and revitalising mental health plans at the district level.

Recent program and policy reviews of RKSK have found that despite substantial investment in clinical service delivery across the health system, management structures, and community engagement, there are problems with the quality of services and human resources (Barua et al., 2020; Roy et al., 2019) particularly training, supporting, and retaining peer-educators. A review (Siddiqui et al., 2020) of 13 non-randomised and randomised evaluations of peer education initiatives to promote sexual and reproductive health in India concluded that although the effects on young people's knowledge, attitudes and behaviours are variable, the peer education model holds promise for the overall canon of interventions to address the sexual and reproductive health of India's cohort of adolescents. We concur: until the RKSK's peer-education model undergoes rigorous evaluation, its potential for transforming the health of India's adolescents across multiple domains of health will remain untapped.

We recommend future research on iterative programming and evaluations to test interventions on incrementally ambitious steps in the theory of change (knowledge, improved health literacy, behaviour change, and finally outcomes) rather than testing programmes on all these simultaneously with no intermediate opportunity for learning and intervention refinement. India's RKSK remains a potential instrument for integrated facility and community-based programming and is the only existing policy which engages young people in the implementation of health intervention through peer educators.

## Funding

Children's Investment Fund Foundation (grant number G160100937).

## Ethics approval

Ethical approval for the trial was obtained from an independent ethics committee in India convened by Ekjut in May 2016 and January 2020, and from University College London Research Ethics Committee in May 2016 (reference: 2656/002) and January 2020 (reference 7403/002). The trial was overseen by a Data Monitoring Committee.

## Role of the funding source

The funder had no role in study design, data collection, analysis and interpretation, in the writing of the report or the decision to submit the manuscript for publication.

## Patient and public involvement

In our formative research we sought the views of local stakeholders, parents, teachers, and adolescents who provided input on what an adolescent health intervention should include. This allowed the local community to indicate the need to include education as an outcome to widen the remit beyond health, but they were not involved in selecting an outcome indicator. Since we designed a participatory intervention, were able to include adolescents in planning intervention delivery, and each group of adolescents made decisions about the logistics of intervention activities to meet their needs. Plans to include a community-wide dissemination event were curtailed due to COVID-19 lockdown in India, but each participatory adolescent group in intervention clusters was able to conduct its own evaluation exercise to assess whether the intervention had been effective in addressing their health and wellbeing (data not shown).

## Declaration of competing interest

The authors declare that they have no competing interests.

## Data Availability

De-identified participant data and Stata code to reproduce analyses for primary and secondary outcomes are available at a private link https://figshare.com/s/20b46535925e26ea3da2
