# Supplementary material for: Effects of community youth teams facilitating participatory adolescent groups, youth leadership activities and livelihood promotion to improve school attendance, dietary diversity and mental health among adolescent girls in rural eastern India (JIAH trial): A cluster-randomised controlled trial
Source: SSM Popul Health. 2022 Dec 27;21:101330. doi: 10.1016/j.ssmph.2022.101330 (PMC9811248; doi:10.1016/j.ssmph.2022.101330)
Supplement: Multimedia component 1 [file mmc1.docx]

**Supplementary material**

Contents

[S1. CONSORT checklist 2](#_Toc106970859)

[S2. TIDieR (Template for Intervention Description and Replication) Checklist 5](#_Toc106970860)

[S3. Data analysis plan 6](#_Toc106970861)

[S4. Process evaluation plan 22](#_Toc106970862)

[S5. Intervention description 29](#_Toc106970863)

[S6. Revised sample size and power calculation following interruption due to the COVID-19 pandemic 30](#_Toc106970864)

[S7. Comparison of baseline information for full and reduced samples followed up at endline 32](#_Toc106970865)

[S8. Baseline characteristics by study arm 33](#_Toc106970866)

# S1. CONSORT checklist

**Table S1. CONSORT checklist**

| Section/Topic | Item No | Standard Checklist item | Extension for cluster designs | Page No |
| --- | --- | --- | --- | --- |
| Title and abstract | | | |  |
|  | 1a | Identification as a randomised trial in the title | Identification as a cluster randomised trial in the title | 1 |
|  | 1b | Structured summary of trial design, methods, results, and conclusions (for specific guidance see CONSORT for abstracts) | See table 2 | 2 |
| Introduction | | | |  |
| Background and objectives | 2a | Scientific background and explanation of rationale | Rationale for using a cluster design | 6 |
|  | 2b | Specific objectives or hypotheses | Whether objectives pertain to the the cluster level, the individual participant level or both | 6 |
| Methods | | | |  |
| Trial design | 3a | Description of trial design (such as parallel, factorial) including allocation ratio | Definition of cluster and description of how the design features apply to the clusters | 7 |
|  | 3b | Important changes to methods after trial commencement (such as eligibility criteria), with reasons |  | NA |
| Participants | 4a | Eligibility criteria for participants | Eligibility criteria for clusters | 7 |
|  | 4b | Settings and locations where the data were collected |  | 7 |
| Interventions | 5 | The interventions for each group with sufficient details to allow replication, including how and when they were actually administered | Whether interventions pertain to the cluster level, the individual participant level or both | 10 |
| Outcomes | 6a | Completely defined pre-specified primary and secondary outcome measures, including how and when they were assessed | Whether outcome measures pertain to the cluster level, the individual participant level or both | 12 |
|  | 6b | Any changes to trial outcomes after the trial commenced, with reasons |  | NA |
| Sample size | 7a | How sample size was determined | Method of calculation, number of clusters(s) (and whether equal or unequal cluster sizes are assumed), cluster size, a coefficient of intracluster correlation (ICC or *k*), and an indication of its uncertainty | 12 |
|  | 7b | When applicable, explanation of any interim analyses and stopping guidelines |  | NA |
| Randomisation: | | | |  |
| Sequence generation | 8a | Method used to generate the random allocation sequence |  | 8 |
|  | 8b | Type of randomisation; details of any restriction (such as blocking and block size) | Details of stratification or matching if used | 8 |
| Allocation concealment mechanism | 9 | Mechanism used to implement the random allocation sequence (such as sequentially numbered containers), describing any steps taken to conceal the sequence until interventions were assigned | Specification that allocation was based on clusters rather than individuals and whether allocation concealment (if any) was at the cluster level, the individual participant level or both | 8 |
| Implementation | 10 | Who generated the random allocation sequence, who enrolled participants, and who assigned participants to interventions | Replace by 10a, 10b and 10c |  |
|  | 10a |  | Who generated the random allocation sequence, who enrolled clusters, and who assigned clusters to interventions | 8 |
|  | 10b |  | Mechanism by which individual participants were included in clusters for the purposes of the trial (such as complete enumeration, random sampling) | 7 |
|  | 10c |  | From whom consent was sought (representatives of the cluster, or individual cluster members, or both), and whether consent was sought before or after randomisation | 7 |
|  |  |  |  |  |
| Blinding | 11a | If done, who was blinded after assignment to interventions (for example, participants, care providers, those assessing outcomes) and how |  | 8, 14 |
|  | 11b | If relevant, description of the similarity of interventions |  |  |
| Statistical methods | 12a | Statistical methods used to compare groups for primary and secondary outcomes | How clustering was taken into account | 12 |
|  | 12b | Methods for additional analyses, such as subgroup analyses and adjusted analyses |  | 13 |
| Results | | | |  |
| Participant flow (a diagram is strongly recommended) | 13a | For each group, the numbers of participants who were randomly assigned, received intended treatment, and were analysed for the primary outcome | For each group, the numbers of clusters that were randomly assigned, received intended treatment, and were analysed for the primary outcome | 14 |
|  | 13b | For each group, losses and exclusions after randomisation, together with reasons | For each group, losses and exclusions for both clusters and individual cluster members | 2, 14 |
| Recruitment | 14a | Dates defining the periods of recruitment and follow-up |  | 14 |
|  | 14b | Why the trial ended or was stopped |  | 14 |
| Baseline data | 15 | A table showing baseline demographic and clinical characteristics for each group | Baseline characteristics for the individual and cluster levels as applicable for each group | 15 |
| Numbers analysed | 16 | For each group, number of participants (denominator) included in each analysis and whether the analysis was by original assigned groups | For each group, number of clusters included in each analysis | 14, 15 |
| Outcomes and estimation | 17a | For each primary and secondary outcome, results for each group, and the estimated effect size and its precision (such as 95% confidence interval) | Results at the individual or cluster level as applicable and a coefficient of intracluster correlation (ICC or k) for each primary outcome | 16 |
|  | 17b | For binary outcomes, presentation of both absolute and relative effect sizes is recommended |  | 16 |
| Ancillary analyses | 18 | Results of any other analyses performed, including subgroup analyses and adjusted analyses, distinguishing pre-specified from exploratory |  | 17 |
| Harms | 19 | All important harms or unintended effects in each group (for specific guidance see CONSORT for harms) |  | 18 |
| Discussion | | | |  |
| Limitations | 20 | Trial limitations, addressing sources of potential bias, imprecision, and, if relevant, multiplicity of analyses |  | 20 |
| Generalisability | 21 | Generalisability (external validity, applicability) of the trial findings | Generalisability to clusters and/or individual participants (as relevant) | 22 |
| Interpretation | 22 | Interpretation consistent with results, balancing benefits and harms, and considering other relevant evidence |  | 21 |
| Other information | | |  |  |
| Registration | 23 | Registration number and name of trial registry |  | 14 |
| Protocol | 24 | Where the full trial protocol can be accessed, if available |  | 7 |
| Funding | 25 | Sources of funding and other support (such as supply of drugs), role of funders |  | 14, 24 |

# S2. TIDieR (Template for Intervention Description and Replication) Checklist

**Table S2. TIDieR checklist**

| **Item number** | **Item** | **Where located **** | |
| --- | --- | --- | --- |
|  |  | Primary paper  (page or appendix  number) | Other ^†^ (details) |
|  | **BRIEF NAME** |  |  |
| **1.** | Provide the name or a phrase that describes the intervention. | Page 6 | ______________ |
|  | **WHY** |  |  |
| **2.** | Describe any rationale, theory, or goal of the elements essential to the intervention. | Page 6 | Protocol (p1) |
|  | **WHAT** |  |  |
| **3.** | Materials: Describe any physical or informational materials used in the intervention, including those provided to participants or used in intervention delivery or in training of intervention providers. Provide information on where the materials can be accessed (e.g. online appendix, URL). | Pages 10-12 | _____________ |
| **4.** | Procedures: Describe each of the procedures, activities, and/or processes used in the intervention, including any enabling or support activities. | Pages 10-12 | _____________ |
|  | **WHO PROVIDED** |  |  |
| **5.** | For each category of intervention provider (e.g. psychologist, nursing assistant), describe their expertise, background and any specific training given. | Pages 10-12 | _____________ |
|  | **HOW** |  |  |
| **6.** | Describe the modes of delivery (e.g. face-to-face or by some other mechanism, such as internet or telephone) of the intervention and whether it was provided individually or in a group. | Pages 10-12 | _____________ |
|  | **WHERE** |  |  |
| **7.** | Describe the type(s) of location(s) where the intervention occurred, including any necessary infrastructure or relevant features. | Pages 10-12 | _____________ |
|  | **WHEN and HOW MUCH** |  |  |
| **8.** | Describe the number of times the intervention was delivered and over what period of time including the number of sessions, their schedule, and their duration, intensity or dose. | Page 15 | _____________ |
|  | **TAILORING** |  |  |
| **9.** | If the intervention was planned to be personalised, titrated or adapted, then describe what, why, when, and how. | Pages 15-16, Supplementary File S5 | _____________ |
|  | **MODIFICATIONS** |  |  |
| **10.^ǂ^** | If the intervention was modified during the course of the study, describe the changes (what, why, when, and how). | NA | _____________ |
|  | **HOW WELL** |  |  |
| **11.** | Planned: If intervention adherence or fidelity was assessed, describe how and by whom, and if any strategies were used to maintain or improve fidelity, describe them. | _____________ | Protocol (p5) |
| **12.^ǂ^** | Actual: If intervention adherence or fidelity was assessed, describe the extent to which the intervention was delivered as planned. | Page 16 | _____________ |

# S3. Data analysis plan

**JHARKHAND INITIATIVE FOR ADOLESCENT HEALTH TRIAL**

**TRIAL STATISTICAL ANALYSIS PLAN**

**Aim of the trial and research questions**

We aim to assess whether an intervention involving a Community Youth Team facilitating participatory peer-led adolescent groups, youth leadership training and livelihood promotion can improve school attendance, dietary diversity and mental health among adolescent girls in rural India.

**Primary research questions**

- What is the effect of an intervention comprising participatory adolescent groups, youth leadership training and livelihood promotion, delivered by a Community Youth Team, on the dietary diversity of adolescent girls aged 10- 19?
- What is the effect of the intervention on adolescent girls’ mental health (internalising and externalising symptoms)?
- What is the effect of the intervention on the number of adolescent girls currently attending school?

**Secondary research questions**

- Does the intervention increase adolescent girls’ ability to make decisions independently and with others about friends, spending money and purchases?
- Does the intervention affect adolescent girls’ attitudes towards gender roles related to education and domestic work?
- Does the intervention improve adolescent girls’ self-efficacy and increase their social-emotional assets?
- Does the intervention reduce adolescent girls’ exposure to emotional and physical violence and increase the number of girls that intervened to reduce emotional and physical violence against their peers in the past year?
- Does the intervention increase the uptake of school-related entitlements (e.g. cash, bicycles, books, midday meals for girls in upper primary) and reduce the number of girls who were absent from school in the past two weeks?
- Does the intervention reduce the number of adolescent girls who drank alcohol in the past month?

**Design of the trial**

**Setting**

The study will be located in Jharkhand, a state in eastern India. Within this state we will work in Khuntpani block, West Singhbhum district.

**Participants, eligibility and migration**

**Eligibility for the baseline and endline survey:** Adolescent girls aged 10-19 years who are living in the study area are eligible to participate in the baseline and endline surveys. We will include married and unmarried girls in the surveys, as well as in school and out of school girls, and girls living in the same household. We will exclude girls who are unavailable for interview at the time of the survey because they have either migrated permanently or migrated temporarily and are not available following repeat visits.

**Eligibility to receive the intervention:** Intervention activities are open to all adolescent girls and boys aged 10-19 years living in the study area. This includes adolescents who are married or unmarried and who are in-school or out-of-school. It also includes adolescents who move into or out of the study area during the study. Adolescents themselves determine the extent to which they engage in the intervention activities and therefore exposure will vary at an individual level.

**Intervention and control exposures**

The intervention involves a ‘Community Youth Team’ conducting three parallel activities:

1. **Participatory groups** for adolescent boys and girls, facilitated by peer educators (‘yuva sathis’). These groups meet monthly to work through four consecutive participatory learning and action cycles on themes of health (including mental health), nutrition, education and violence. They identify and address problems associated with each of these themes with the support of the wider community.
2. **Youth leadership training**: Adult facilitators run cultural and sports activities for adolescent boys and girls every two months. Activities are designed to be fun and engaging and to help build adolescents’ confidence and self-efficacy.
3. **Livelihood promotion**: Adolescent boys and girls and their parents who live in intervention and control arms are invited to take part in activities related to farming and environmental management to improve their practical skills and food security. Activities are facilitated by adults and run approximately every three months.

**Unit of randomisation**

The study is a cluster-randomised controlled trial. Clusters are purposively selected areas of approximately 1000 population, comprising a village and its hamlets. Clusters are separated by distance or natural boundaries (e.g. hills and rivers), reducing the risk of exposure to intervention activities in the control arm.

**Randomisation method**

Thirty-eight clusters covering an estimated total population of 40,676 were randomised to control and intervention arms, nineteen to each arm. Clusters were stratified for randomisation based on five strata: (i) clusters with a secondary school and an adolescent club (clubs are community-based health and development groups for adolescents run by the Integrated Development Foundation), (ii) clusters with a secondary school and no adolescent clubs, (iii) clusters without a secondary school but with an adolescent club, (iv) clusters without a secondary school and without an adolescent club, (v) clusters having a population of more than 1500.

**Outcome data collection**

Once an adolescent girl (and her caregiver if the girl is <18 years) has consented to participate in the study, the monitor administers a structured questionnaire including questions on health, nutrition, gender norms and equity, mental health, wellbeing, and education. The monitor also measures the girl’s height, weight and mid upper arm circumference (MUAC). The data collection process is the same at baseline and endline, though some additional questions have been added in the endline survey. Additional qualitative and quantitative data will be collected for the process evaluation however these data will not be considered in this analysis plan.

**Primary, secondary and tertiary outcome measures**

Outcome measures are presented in Table 1. We set out a priori to address the three most important outcomes identified through our needs assessment and the intervention’s theory of change.

**Table S3: Primary, secondary and tertiary outcome measures**

| **Primary outcomes** | | **Source of data** | **Baseline data available?** | **Type of outcome** | **Details of tools, measurements or questions** | **Method for deriving analysis variable(s)** |
| --- | --- | --- | --- | --- | --- | --- |
| 1 | Mean dietary diversity score, based on 24h recall | Endline survey | Yes | Continuous outcome | We will calculate a dietary diversity score based on 10 food groups:   1. Grains and tubers: roti, rice, peetha, other goods made from grains, mudhi/Food made from roots 2. Pulses: soya bean, other beans, peas, lentils 3. Nuts and seeds: Sunflower seeds, dori, mahua, kudrum, tisi, ramtia, linseed, sesame, chahar, mustard 4. Dairy: Cheese, yoghurt or other milk products 5. Meat, poultry and fish: liver, kidney, heart or other organ meat, chicken, duck or other birds, pork, lamb or goat, fresh fish, dried fish, shellfish or seafood, grubs, snails or insects 6. Egg 7. Dark green leafy vegetables 8. Other vitamin A-rich fruits and vegetables: pumpkin, carrot, sweet potato (orange/yellow inside)/Ripe mango, ripe papaya, ripe jackfruit 9. Other vegetables 10. Other fruits/Unripe mango, unripe papaya, unripe jackfruit | Responses will be coded 0/1 (No/Yes) for each food group.  Each participant’s Dietary Diversity Score will be calculated as the sum of number of food groups consumed. All food groups are equally weighted.  Scores will range from 0 to 10, with higher score indicative of greater dietary diversity.  [NB: the binary classification for ‘minimum’ dietary diversity has a cut-off of 5 out of 10] |
| 2 | Mean score on the Brief Problem Monitor - Youth | Endline survey | BPC data only* | Continuous outcome | Brief Problem Monitor (BPM)-Youth* questions, with possible edits following validation:   1. I act too young for my age 2. I argue a lot 3. I fail to finish things I start 4. I have trouble concentrating or paying attention 5. I have trouble sitting still 6. I destroy things belonging to others 7. I disobey my parents 8. I disobey at school 9. I feel worthless or inferior 10. I act without stopping to think 11. I am too fearful or anxious 12. I feel too guilty 13. I am self-conscious or easily embarrassed 14. I am inattentive or easily distracted 15. I am stubborn 16. I have a hot temper 17. I threaten to hurt people 18. I am unhappy, sad, or depressed 19. I worry a lot 20. I am easily annoyed / irritable 21. I am often unwell | Responses will be coded 0= Not true, 1 = Somewhat true, 2= Very true.  Omitted responses will count as 0.  Each participant’s total score on the BPM-Y will be calculated as the sum of responses to 20 out of 21 items (excluding item 8), out of a total of 40. Higher scores will indicate greater problems.  The 3 sub-scales of the BPM-Y will be analysed separately, and will be categorised as follows:  Internalising: Items 9, 11, 12, 13, 18, 19  Externalising: Items 2, 6, 7, 15, 16, 17  Attention: Items 1, 3, 4, 5, 10, 14 |
| 3 | % of adolescent girls attending school or college | Endline survey | Yes | Dichotomous outcome | - Do you currently attend a school or college? | Responses will be coded 0 / 1 for No / Yes |
| **Secondary outcomes** | |  |  |  |  |  |
| 1 | % of girls making decisions independently and with others about the food they eat including how much they eat and what types of food they eat | Endline survey | Yes | Dichotomous outcome | - Who usually makes decisions about the food that you eat, including how much you eat and what types of food you eat? (Read options and select more than one option if appropriate) | Response checklist items will be summarised for each individual to produce a binary variable describing decision-making methods coded 0= Respondent only / others only decide  1= Adolescent decides jointly with others |
| 2 | Mean score on gender role attitudes index | Endline survey | Yes | Continuous outcome | Gender role index:   1. Do you think that educating boys is more important than educating girls? 2. Do you think that girls are usually as good as boys in studies? 3. Do you think that boys should do as much domestic work as girls? 4. Do you think that girls who are teased deserve it if they are dressed provocatively? 5. Do you think that a woman should obtain her husband’s permission for most of the things? 6. Do you think that girls should be allowed to decide when they want to marry? 7. Do you think that the husband alone/mainly should decide how household money is to be spent? | Responses are coded 0 /1 (No / Yes) for each question, with reverse coding applied at the data management stage for questions 1, 4, 5, 7.  Each participant’s responses are summed to derive a total score out 7, with higher scores indicating greater gender egalitarian attitudes. |
| 3 | % of girls making decisions independently and with others about friends, spending money and purchases | Endline survey | Yes | Dichotomous outcome | 1. Who mainly takes the decision about who your friends would be? 2. Who mainly takes the decision about how to spend your money? 3. Who mainly takes the decision about what you buy? | For each question, checklist responses will first be summarised as a binary variable to describe decision-making methods as 0= Respondent only / others only decide and 1= Adolescents decide jointly with others.  Responses will be summed to derive a total score out of 3, and subsequently dichotomised as  0= Total score below 3  1= Total score of 3 |
| 4 | Mean score on the Schwarzer General Self-Efficacy (GSE) Scale | Endline survey | No | Continuous outcome | GSE Scale:   1. If someone opposes me, I can find the means and ways to get what I want. 2. It is easy for me to stick to my aims and accomplish my goals. 3. I am confident that I could deal efficiently with unexpected events. 4. Thanks to my resourcefulness, I know how to handle unforeseen situations. 5. I can remain calm when facing difficulties because I can rely on my coping abilities. 6. I can usually handle whatever comes my way. | Response options are coded 1= Not at all true, 2= Somewhat true, 3= Exactly true.  Total GSE score is calculated as the sum of all items.  Total scores range between 6 and 18 points, with higher scores indicating greater self-efficacy. |
| 5 | Mean score on the Child and Youth Resilience Measure –Revised 10-item version (CYRM-R) | Endline survey | No | Continuous outcome | 1. I cooperate with people around me 2. Getting an education is important to me 3. I know how to behave in different social situations 4. People like to spend time with me 5. I feel supported by my friends 6. I feel I belong at my school 7. My friends stand by me in difficult times 8. I am treated fairly in my community 9. I have opportunities to show others that I am becoming an adult and can act responsibly 10. I have opportunities to develop skills that will be useful later in life (like job skills and skills to care for others) | Response options are coded 1= No, 2= Sometimes, 3= Yes.  Total CYRM-score is calculated as a sum of all items. Items are equally weighted.  Total scores range between 10 and 30, with higher scores indicating greater resilience.  Item non-response can be dealt with using imputation methods. |
| 6 | % of girls who report experiencing emotional violence in the past 12 months | Endline survey | Yes | Dichotomous outcome | Questions relating to emotional violence   - Has anyone ever: Cursed or insulted you, called you rude or hurtful names? - Has anyone ever: Humiliated or belittled you in front of other people, or embarrassed you? - Has anyone ever: Done things to scare or intimidate you on purpose, or threatened to hurt someone you care about ? - Has anyone ever: Forced you to stay inside or outside - Have you been made to work/look after siblings when you wanted to go to school? | Response options for each question are coded 0= No, 1= Yes.  The outcome will be coded 1= Yes if the participant has experienced any type of emotional violence, and 0= No if they have not experienced any in the past year. |
| 7 | % of girls who report experiencing physical violence in the past 12 months | Endline | Yes | Dichotomous outcome | Questions relating to physical violence   - Has anyone ever: Twisted your arm or any other body part, slapped you, pushed you, pulled your hair or thrown something at you? - Has anyone ever: Threatened to use or actually used a gun, knife or other weapon against you? - Has anyone ever: Choked you on purpose, dragged or beaten you up, tied you up with a rope or belt? - Has anyone ever: Punched you, kicked you, hit you with a closed fist or hit you with an object, such as a stick or a cane, or whipped you? - Has anyone ever: Severely beaten you up, cut you with a sharp object or burnt you purposefully? | Response options for each question are coded 0= No, 1= Yes.  The outcome will be coded 1= Yes if the participant has experienced any type of physical violence, and 0= No if they have not experienced any. |
| 8 | % of girls who report intervening to reduce emotional violence against their peers in the past 12 months | Endline survey | No | Dichotomous outcome | (Questions correspond to each type of emotional violence described in questions for outcome 2.6) | Response options for each question are coded 0= No, 1= Yes.  The outcome will be coded 1= Yes if the participant reports intervening to stop any type of emotional violence against their peers, and 0= No if they report they have not. |
| 9 | % of girls who report intervening to reduce physical violence against their peers in the past 12 months | Endline survey | No | Dichotomous outcome | (Questions correspond to each type of physical violence described in questions for outcome 2.7) | Response options for each question are coded 0= No, 1= Yes.  The outcome will be coded 1= Yes if the participant reports intervening top stop any type of physical violence against their peers, and 0= No if they report they have not. |
| 10 | % of girls who report being absent from school in the past two weeks | Endline survey | Yes | Dichotomous outcome | - In the past two weeks did you have to miss any days of school/college? (not including holidays or weekends) | Responses coded 0= No, 1=Yes |
| 11 | % of girls accessing at least one school-related entitlement (cash, bicycles, books, midday meal scheme) | Endline survey | No | Dichotomous outcome | Which school entitlements did you get in the last year? [participants select all that apply]   - Cash - Bicycle - Books - Midday Meal - None | Responses coded 0= None, 1=Yes to one or more (cash, bicycle, books, midday meal). |
| 12 | % of girls who drank alcohol in the past month | Endline survey | Yes | Dichotomous outcome | - Have you ever had any alcohol, other than a few sips? - During the last 1 month how often did you drink more than a few sips? | The first question will be coded 0=No, 1=Yes.  The second question will be coded 0= Not in last 1 month, 1= Once a month / fortnight / week or every day / other day.  The outcome will be coded 1 =Yes if the participant’s answer to both questions is 1, and 0= No if it is 0 for the first question and N/A to the second (skip constraint) OR 1 for the first question but 0 for the second. |
| **Tertiary outcomes** | |  |  |  |  |  |
| 1 | % of girls who took at least four iron and folic acid supplements in the past month | Endline survey | Yes | Dichotomous outcome | In the past 1 month, have you received any iron and folic acid tablets?  How many iron and folic acid tablets did you consume in the past month? | Response to first question coded as 0=No, 1=Yes (next question skipped if response=0).  Response to second question recorded as integer reported by adolescent.  Data will be dichotomised at 4 (0= Fewer than 4 tablets; 1= 4 or more tablets). |
| 2 | % of girls aged 15-19 and all married girls who have correct knowledge about the contraceptive pill, condoms and the IUD | Endline survey | Yes | Dichotomous outcome | - How often should a woman take oral pills? - One condom can be used for how many acts of sexual intercourse? - Where is the IUD placed? | Analysis will be combined for both groups (15-19 years and married adolescents of any age), and the total number of adolescents in these groups will form the denominator for this outcome.  Response options for each question are coded 0= Incorrect, 1 = Correct.  The outcome will be coded 1= Yes if participants give the correct answer to all three questions, and 0=No otherwise. |
| 3 | % of girls who use sanitary napkins or clean cloths during their period | Endline survey | Yes | Dichotomous outcome | Girls can use different methods for protection during their menstrual period to prevent bloodstains from becoming apparent. What do you mainly use for protection, if anything?   - Single use sanitary pad - Single use cloth (home-made or from a shop) - Cloth that they wash and re-use - Other method - Nothing | Responses will be coded 0= No (Cloth that they wash and re-use, other method, nothing) and 1= Yes (Single use sanitary pad or single use cloth (home-made or from a shop)). |
| 4 | % of girls aged 15-19 and all married girls who know that abortion is legal | Endline survey | Yes | Dichotomous outcome | If any woman has an unwanted pregnancy and wants to terminate it, is it legal for her to abort it? | Analysis will be combined for both groups (15-19 years and married adolescents of any age), and the total number of adolescents in these groups will form the denominator for this outcome.  Responses coded 0= No, 1=Yes |
| 5 | % of girls who have received take home rations in the past month | Endline survey | Yes | Dichotomous outcome | In the past 1 month did you receive any Take Home Rations (THR) that were for you? | Responses coded 0= No, 1=Yes |
| 6 | % of girls underweight (<-2SD median BMI for age and  sex) | Endline survey | Yes | Dichotomous outcome |  | Calculated using the WHO Growth Standards, using the ‘zanthro’ add-on in Stata |
| 7 | % of girls stunted (<-2SD median height for age and  sex) | Endline survey | Yes | Dichotomous outcome |  | Calculated using the WHO Growth Standards, using the ‘zanthro’ add-on in Stata |
| 8 | Mean MUAC score | Endline survey | Yes | Continuous outcome |  | Measured in centimetres |

* The BPC used at baseline has been replaced for the endline survey by the Brief Problem Monitor – Youth, which has a six-item Attention Problems scale in addition to Internalising, Externalising and Total Problems scales

**Sample size calculation**

The size of the study area was chosen for logistical reasons. With a district-level crude birth rate of around 23 per 1000 population and accounting for child deaths, we expected to find an estimated 8800 adolescents aged 10-19 (4400 girls) in our proposed intervention area. We anticipated that villages and their adjoined hamlets would have an average population of c.1000 each. In each village, there would be c.220 adolescent boys and girls aged 10-19 years.

3324 adolescent girls aged 10-19 years participated in the baseline survey: 82% of an estimated 4068 girls in the 38 clusters. The mean number of girls in each cluster was 87 (standard deviation 29.9). Assuming we are able to interview the same number of girls in the endline survey, with a mean cluster size of 87 and a coefficient of variation of cluster sizes of 0.3, the trial will have 80% power to detect: a nine percentage point increase in the proportion of girls attending school or college (ICC 0.03), from a baseline prevalence of 69% to 78%; a 0.9 point increase in mean dietary diversity score (ICC 0.40), from a mean baseline score of 3.4 (SD 1.4) to 4.3; a 2.7-point decrease in Brief Problem Monitor - Youth score (ICC 0.39) from a mean baseline score of 6.0 (SD: 4.3) to 3.4, all at a significance level of 0.05. We performed these calculations in Stata (version 14). We anticipate that including baseline data in our analysis will lead to gains in power but do not attempt to quantify these here.

**Approach to final analysis**

**General principles**

Our main analyses will be by **intention to treat**, meaning that analyses will be carried out based on the groups as randomised, regardless of their level of intervention exposure. As described in our Subgroup Analyses section, we will also assess whether the intervention effect differs according to intervention exposure.

We have three primary outcomes. We think that success in improving at least one of these outcomes could help inform decisions about future scale up. We will declare the trial a success if we find a significant (two-tailed p<0.05) benefit for at least one outcome, in conjunction with a collectively ‘positive signal’ for the other two outcomes. A ‘positive signal’ is defined as at least one of the two outcomes in the direction of benefit, neither outcome showing significant harm, and if for one outcome the direction of effect is towards benefit and for the other it is towards harm then we require the (two-tailed test) p-value for the former outcome to be smaller than that for the latter. The type I error rate corresponding to this definition of success varies according to the correlations between the three outcomes, which we expect to be positive. Under perfect positive correlations the type I error rate is 5%. Under independence and the null hypothesis (no intervention effect on any outcome) the probability of observing success in conjunction with 1, 2 or 3 significant benefits observed for individual outcomes is 3.38% (3 x 0.025 x 0.95^2^ x 0.5), 0.18% (3 x 0.025^2^ x 0.95), and 0.00% (0.025^3^) respectively. Consequently the overall probability is 3.56% and the type I error rate assuming a symmetric definition of overall trial failure (harm) is 7.12%.

For primary and secondary outcomes, we will conduct analyses to compare differences between the control and intervention arms of the trial, using regression models with Generalised Estimated Equations (GEE) to adjust for clustering. We will model baseline and endline information together, but this will be ‘cross-sectional’ analysis, in the sense that we will not match individuals in the two surveys. For the BPM-Y outcome we will model the data at endline jointly with the BPC data collected at baseline. We will use the exchangeable working correlation structure. To assess the effect of the intervention our regression models will include an indicator of time (endline vs. baseline) and an indicator of intervention, coded one for participants measured at endline in the intervention arm and zero otherwise. This analysis approach has been termed a constrained baseline analysis.

Our baseline data show that dietary diversity is slightly positively skewed, and BPM-Y scores are very positively skewed. We will model mean dietary diversity scores using linear regression. We will model BPM-Y scores using linear regression, after transformation by the function log(1+x), noting that our analysis approach (marginal model fitted by GEE) provides robustness by avoiding distributional assumptions. We will use a logistic regression model to test for a difference in the % of girls attending school between arms as a binary outcome.

We will present 95% confidence intervals and consider statistical significance for any outcome to arise if the two-tailed p-value is less than 0.05. Although we have three primary outcomes, as explained earlier we have pre-specified a strategy for deciding whether there is an overall statistically significant benefit of the intervention based on all three outcomes.

Analysis of secondary outcomes will follow the same principles as we have described for the primary outcomes, using linear or logistic regression as appropriate, but we note that some outcomes were not collected at baseline. For such outcomes the endline data will be modelled using GEE including terms for intervention arm and the adjustment factors.

Tertiary outcomes will be reported in the process evaluation as exploratory analyses.

**Blinding**

The researcher conducting the final trial analyses will be blind to allocation, reporting results for “Arm A” with “Arm B”. An independent statistician, also blinded to allocation, will repeat the analyses for the primary outcomes.

**Adjustment**

For each outcome we will adjust for the same set of pre-specified prognostic factors (asset quintile, tribal status and age), and for strata. We will additionally adjust for further socio-economic factors should the Data Monitoring Committee note important imbalances between arms among baseline participants. However, since there could be collinearity between such factors or between such factors and the pre-specified prognostic factors, it may be that not all can be included or that some may need to combined into single factors. If there is substantial missing data for a factor then it will not be included in the model selection (see below). The final model of factors to adjust for will be selected without reference to intervention effect, i.e. without seeing the impact of different potential models on the intervention effect estimate. Adjusted effect measures will be considered primary, but unadjusted effect measures will also be presented for completeness.

**Subgroup and per-protocol analyses**

For each of the primary outcomes, we plan to conduct three sub-group analyses. The first will assess the effects of the intervention on the primary outcomes by age, categorising girls as younger (10-14 years) or older (15-19 years). For school attendance, Brief Problem Monitor score and dietary diversity score, we anticipate the effect of the intervention to be larger among older girls. The second subgroup analysis will examine intervention effects on the primary outcomes for the two poorest wealth quintiles, and for the other quintiles grouped. Based on previous studies of equity impact of participatory learning and action groups in eastern India, we anticipate girls in the two lowest wealth quintiles will receive greatest benefit from the intervention. The third subgroup analysis, alternatively viewed as a ‘per-protocol’ analysis, will test intervention effects by different levels of exposure to the intervention activities. We will create a dichotomous variable to identify high and low intervention exposure. We will define high exposure as having attended at least 15 out of 31 participatory group meetings and at least 8 out of 16 youth leadership meetings. Low exposure will be defined as having attended less than 15 participatory group meetings and less than eight youth leadership meetings. All subgroup analyses will follow the analysis approach of the main analyses with regard to model types and adjustment. The intervention effects on the primary outcomes will be presented within each subgroup, for the third subgroup analysis the exposure groups will be compared to all control arm participants. We will formally test for differences in intervention effects between the two wealth quintile groups, and by age group, by testing an interaction term. The test for an effect of level of exposure on each primary outcome will be conducted by comparing intervention levels in an analysis restricted to the intervention arm participants.

**Treatment of missing data**

We do not plan to conduct any sensitivity analysis into the effects of survey refusal as the refusal rate to the endline survey is expected to be very low, in line with the baseline survey.

Analysis of each primary outcome will be based on available cases if the rate of missing data for the outcome, among those participants who have provided other outcomes, is 10% or less. If missing data rates are higher than 10% we will impute missing values for outcomes using other outcomes as well as other characteristics. Any imputation will be conducted based on the chained equations approach using the mi suite of functions in Stata, and conducted separately by trial arm. The clustering of the data will be ignored for simplicity. If imputation is conducted then the analysis of the imputed data will be seen as primary. Analysis of secondary outcomes will be based on available cases irrespective of the level of missing data. For tools with user guidance documents that outline methods for dealing with item non-response (example, impute or code as 0), we will follow the tool developer’s recommendations.

If there is substantial missing data (>10%) in a factor considered for adjustment then it will not be considered further unless an important imbalance is seen between arms and there are no other factors with little missing data that are clearly correlated with the factor.

**Interim analyses and data monitoring committee**

We will not conduct interim analyses of the endline data. A data monitoring committee will (i) examine the comparability of trial arms and potentially identify socio-economic variables that differ between arms to adjust for in the final analysis; (ii) approve our data analysis plan and ensure we conduct the final analyses in accordance with this; and (iii) provide recommendations for additional analyses.

**Tables and figures for primary publication**

The publication will include a CONSORT diagram, which will show the number of clusters and participants at each stage of the trial. We will include a table of socioeconomic characteristics of participants by trial arm at endline, presenting summary statistics such as means and standard deviation or frequencies and percentages. Characteristics included in this table will be age group and mean age, tribal status, literacy, marital status, asset quintile and household location (village or hamlet). We will present a table of uptake of the intervention and control exposures by trial arm. We will also include a table that presents summary statistics of the primary outcomes by trial arm (median and inter-quartile range, percentage as appropriate) and by baseline vs. endline, with associated effect measures for the intervention, 95% confidence intervals and p values, with and without adjustment. A similar table will also be constructed for secondary and tertiary outcomes.

**Data collection and management**

Trial data will be collected in face-to-face interviews with eligible adolescent girls for individual-level variables and from a parent / caregiver / guardian for household-level information such as household assets or socioeconomic position. Data will be entered in smartphones programmed with CommCare software. The electronic survey form will include skip-constraints and validation checks tailored to specific questions and measures. Data will be stored on the CommCare cloud-server during data collection, with daily updates as data monitors complete interviews and upload forms to CommCare at the end of each working day. Each adolescent participant’s individual responses will be matched to their household-level data in the endline survey and merged into one observation. All responses to the endline survey will be downloaded as Stata datasets at the end of data collection for further data management.

Baseline data will be appended to the endline dataset, with the two surveys coded 0=baseline and 1=endline in a variable indicating time. Cluster numbers will be consistent across the two surveys, but participant ID will not. For example, there could be participants with ID number 27 in several clusters, or there could be observations with the same ID and cluster number at both time points representing different individuals. Unique individuals will be identified by the combination of cluster, ID and time variables. Allocation, outcome and adjustment variables will be common to both, except for outcomes that are only measured at baseline. A .do file with code to derive analysis variables will accompany the trial dataset, with separate versions to comply with requirements for blinded analyses.

Basic data checks are performed by the trial Data Manager at Ekjut periodically during the trial. Additional range, consistency and missing data checks will be performed by the statistician before the statistical analysis is performed. All variables will be examined for unusual, outlying, unlabelled or inconsistent values.

Any problems with trial data will be queried with the Data Manager. If possible, data queries will be resolved; although it is accepted that due to administrative reasons and data availability a small number of problems will continue to exist. These will be minimised.

When trial data collection is complete the trial data manager will save a ‘frozen’ copy of the trial dataset, but with cluster randomisation status masked and codes blinded, and information such as intervention exposure that would unblind the analyst removed. A statistician will then conduct all planned analyses of the primary outcome and secondary outcomes to be evaluated by trial arm. A second statistician will then replicate the analysis. Once these analyses are complete, the Data Manager will create a second frozen copy of the main study dataset for which the cluster randomisation status is not blinded, for the statistician to generate full reporting of all trial outcomes.

If necessary, data can be added to or amended in the main, unfrozen, copy of the dataset.

- If any outstanding queries are resolved during preparation of the initial blinded analysis that relate to data in the frozen dataset (e.g. problems that are found during analysis or notifications of data amendments), the changes need only be made to the main dataset and an updated frozen copy made available.
- If any outstanding data queries are resolved during the unblinded analysis, the main and frozen dataset should both be altered.

An anonymised trial dataset reflecting the data after any amendments have been made with data on primary and secondary outcomes and characteristics used in adjustment, together with code used in the main analyses, will be made available as supplementary files with the main trial publication.

# S4. Process evaluation plan

**AIM**

To understand the context, implementation and mechanisms of impact of an intervention involving community youth teams facilitating participatory learning and action with groups of adolescents, youth leadership and livelihood promotion

**OBJECTIVES**

1. To understand how contextual factors influence intervention implementation and mechanisms of impact
2. To describe intervention implementation including the process of delivery (what resources were used), fidelity (what was planned versus what was implemented), exposure (how many and how regularly did adolescents attend intervention activities) and reach (who attended and who didn’t attend intervention activities)
3. To identify potential mechanisms of impact of the intervention
4. To identify factors that could facilitate or prevent the scale up of the intervention

**DATA SOURCES**

***Quantitative data***

- Baseline survey – village, household, adolescents
- Endline survey (including M&E data on intervention activity attendance)
- Intervention cost data

***Qualitative data: Interviews/focus groups***

- Focus group discussions with yuva sathis at the end of the intervention/at the end of mini PLA cycles
- Focus group discussions with youth leadership facilitators at the end of the intervention
- Focus group discussions with livelihood promoters at the end of the intervention
- Focus group discussion with PLA coordinators and supervisors at the end of the intervention
- In-depth case studies of adolescent boys and girls, their parents and teachers (including those with high participation and those with low or no participation) over two years of intervention
- Interviews with frontline health workers at the end of the intervention

***Reports/notes/forms***

- Participatory group meeting reports (completed by yuva sathis, detailing location, number of group participants, register, topic of meeting)
- Youth leadership training reports (completed by youth leadership facilitators, detailing location, number of participants, register, topic of meeting)
- Livelihood promotion activity reports (completed by livelihood promoter, detailing location, number of participants, register, topic of meeting)
- Prioritised problem and strategy form for each mini PLA cycle (completed by yuva sathis for their group, includes notes on evaluation)
- Community meeting reports (completed by yuva sathis for their group, detailing number of attendees, etc)
- Supervision meeting reports (completed by PLA coordinators and supervisors)
- Education/nutrition entitlements mapping by yuva sathis

***Documents***

- Trial protocol
- PLA meeting plan
- Youth leadership activity plan
- Livelihood promotion meeting plan
- Policy documents

**Table S4: Process evaluation plan**

| **Questions** | **Data Collection Methods** | **Data Source** | **Data analysis** | **Frequency of data collection** | **Timeline** |
| --- | --- | --- | --- | --- | --- |
| ***Objective 1: To understand how contextual factors influence intervention implementation and mechanisms of impact*** | | | | | |
| How does the profile of the intervention and control communities (SC/ST/OBC, literacy, education, socio-economic status, food security) influence the effect of the intervention? | Baseline and endline surveys – adolescent  FGDs with yuva sathis (n=2), youth leadership facilitators (n=1), livelihood promoters (n=1) and PLA coordinators and supervisors (n=1) | Household Module  Adolescent Module  Village Module | Cluster level regression analysis to identify predictors of intervention effect  Participatory analysis using the framework method | Once | Endline survey  Dec 2019-April 2020  Oct 2019-Feb 2020 (data collection)  March -April (Transcription)  May (Analysis)  June 2020 (reporting) |
| What cluster level factors associated with infrastructure and health/education service provision (availability of nutrition and educational entitlements, NGO activities [e.g. adolescent clubs] and government schemes, proximity to secondary school, availability of frontline health workers) are associated with the trial outcomes? | Baseline survey – village level, and baseline and endline surveys - adolescent  FGDs with yuva sathis, youth leadership facilitators, livelihood promoter and PLA coordinators and supervisors  Mapping of education/nutrition and health entitlements for adolescents by PLA coordinators and Supervisors | Household Module  Adolescent Module  Village Module  FGD Transcripts  PLA coordinators and Supervisors consultation meeting notes | Cluster level regression analysis to identify predictors of intervention effect  Participatory analysis using the framework method  Quantitative/qualitative review | Once  Once  Once | Endline survey Jun Dec 2019-April 2020  Oct 2019-Feb 2020 (data collection)  March -April (Transcription)  May (Analysis)  June 2020 (reporting) |
| What were the enablers and barriers to community youth teams implementing the intervention? | FGDs with yuva sathis, youth leadership facilitators, livelihood promoter and PLA coordinators and supervisors  Review of youth leadership facilitators, livelihood promoter and PLA coordinators and supervisors workplans and attendance sheets | FGD Transcripts | Participatory analysis using the framework method  Quantitative review of workplans and attendance sheets | Once | Oct 2019-Feb 2020 (data collection)  March -April (Transcription)  May (Analysis)  June 2020 (reporting)  May 2020 |
| ***Objective 2: To describe intervention implementation including the process of delivery, fidelity, exposure and reach*** | | | | | |
| ***Process of delivery:*** | | | | | |
| How were members of the community youth teams recruited and trained? | Document Review | Training Reports  Recruitment report | Review | Once | Apr/May 2017 |
| What were the topics of the group meetings, youth leadership training and livelihood promotion activities implemented? | Document review | Intervention Modules  PLA Meetings Plan | Review | Once | Mar 2020 |
| Participatory group characteristics (location, number of meetings, timing/date of meetings, size, number of male and female participants) | Document review | JIAH – MIS | Review | Once | Mar 2020 |
| What were the problems and strategies that were prioritised by the participatory groups? | Document review | Prioritised problem and strategy form for each mini PLA cycle | Review | Once | Mar 2020 |
| Youth leadership activities (location, number of activities, timing/date)? | Document review | Youth leadership activities- JIAH -MIS | Review | Once | Mar 2020 |
| Livelihood training activities (location, number of activities, timing/date) | Document review | Livelihood promotion activities – JIAH-MIS | Review | Once | Mar 2020 |
| To what extent and how did the community youth team engage with schools? | Structured interviews (closed and open questions) with teachers from the secondary schools  Document review  Document review  Document review | Structured interviews  Review meeting notes  Strategies implementation records  Prioritised problem and strategy form for mini PLA cycle on education  Wider dissemination meeting reports | Participatory analysis using the framework method  Qualitative review  Qualitative review | Once  End of each mini PLA cycle  Ongoing | Done |
| To what extent and how did the community youth team engage with frontline health workers (AWW, ANM, ASHA)? | Interviews with frontline health workers (two ANMs, two ASHAs, two AWWs)  Document review | SSI Transcripts  Review meeting notes  Strategies implementation records  Prioritised problem and strategy form for each mini PLA cycle  Wider dissemination meeting reports | Participatory analysis using the framework method | Once  Once | Data collection: Jan 2020  May 2020 |
| To what extent and how was the wider community supportive of the intervention activities? | Focus group discussions with yuva sathis, youth leadership facilitators and livelihood promoters  Document review  Document review | FGD Transcripts  Long table attendance records  Community meeting reports  Prioritised problem and strategy form for each mini PLA cycle | Participatory analysis using the framework method  Quantitative/qualitative review  Qualitative review | Once  End of each mini PLA cycle | May 2020 |
| How were yuva saathis supervised and how successful was this? | Focus group discussions with yuva sathis and PLA coordinators and supervisors | FGD Transcripts  Supervision meeting reports | Participatory analysis using the framework method  Quantitative/qualitative review | Once  Ongoing | Mar 2020 |
| What were the characteristics, attributes and skills of yuva saathis, youth leadership facilitators and leadership coordinators and how did this impact on their competence to facilitate the participatory group meetings? | In-depth interviews with 6 adolescents boys and 6 girls, their parents and teachers (14-19 years), for case studies  Review meeting  Assessment  (Training and field) | Interview transcripts  Visit Notes  Review meeting notes  Assessment scores (Training and field) | Participatory analysis using the framework method  Quantitative review  Quantitative/qualitative review | Once  Once  Ongoing | Aug 2019-May 2020 |
| ***Fidelity*:** | | | | | |
| How many intervention activities were postponed/discontinued and why were they postponed? | Document review | PLA meeting postponement format/Youth leadership activity plan/Livelihood promotion activity plan | Quantitative/qualitative review | Ongoing | May 2020 |
| ***Exposure:*** | | | | | |
| How many adolescent girls and boys aged 10-19 attended the participatory group meetings, youth leadership and livelihood promotion activities? | Document review | JIAH – MIS Formats | Quantitative analysis | Ongoing | Mar 2020 |
| How many intervention activities did adolescent girls attend? | Endline survey – adolescents | Household Module  Adolescent Module  Village Module | Quantitative analysis | Once | Dec 2019-April 2020 |
| What were the enablers and barriers to girls and boys attending the intervention activities? | In-depth case studies and interviews with 6 adolescents boys and 6 girls, their parents and teachers (14-19 years) | FGD Transcripts | Participatory analysis using the framework method | Once | Aug 2019-May 2020 |
| ***Reach:*** | | | | | |
| What was the composition of adolescents at participatory group meetings, youth leadership training and livelihood promotion activities (number, member characteristics [number of younger versus older adolescents, married, sex,in/out of school])? | Document review | JIAH – MIS Formats | Quantitative review | Ongoing | Mar 2020 |
| ***Objective 3: To identify potential mechanisms of impact of the intervention*** | | | | | |
| What are the potential changes at the individual, family and community level in terms of learning, motivation and action? | Group discussion with intervention team members to review the process evaluation findings and endline findings alongside the theory of change | Discussion Minutes |  | Once | June 2020 |
| ***Objective 4: To identify factors that could facilitate or prevent the scale up of the intervention*** | | | | | |
| How could the community youth team be integrated into existing community health and education systems? | Stakeholder consultation/meetings  Interviews with frontline health workers  Focus group discussions with teachers  Document review | Stakeholders discussion notes  Interview transcripts  FGD transcripts  RKSK meeting  Policy documents | Qualitative review of notes  Participatory analysis using the framework method  Participatory analysis using the framework method | Once  Once  Once | Jan 2020-May 2020  Jan 2020-May 2020  Jun 2020 |
| What are the operational requirements to scale up the intervention? | Stakeholder consultation/meetings | Discussion Notes | Qualitative review of notes | Once |  |
| What are the HR requirements to scale up the intervention? | Stakeholder consultation/meetings | Discussion Notes | Qualitative review of notes | Once |  |
| What is the cost of the intervention? | Intervention costing data | Accounts statement | Quantitative modelling | Once |  |

# S5. Intervention description

In the first phase of each PLA cycle, *yuva saathi*s used picture cards depicting problems that adolescents might face. These picture cards illustrated the following problems:

**Table S5: Description of problem picture cards used in PLA mini-cycles**

| Education | Gender norms related to education |
| --- | --- |
|  | School drop-out |
|  | Lack of access to school-related entitlements |
| Nutrition | Anaemia |
|  | Lack of access to nutrition-related entitlements |
|  | Inadequate dietary intake and dietary diversity |
|  | Intra-household food distribution |
|  | Intra-household food insecurity |
| Health | Lack of menstrual hygiene and menstrual disorders |
|  | Early marriage and adolescent pregnancy |
|  | Alcohol and substance abuse |
|  | Depression and anxiety |
|  | Behavioural disorders |
|  | Lack of access to health entitlements |
| Violence | Street harassment |
|  | Physical and emotional violence |
|  | Sexual harassment |
|  | Not being able to voice their opinions |

Each group voted on three problems they would like to address, and selected one or two for further discussion. Some adolescents identified problems that were not discussed in the picture cards. Problems mentioned by adolescents that were not represented on the picture cards are written down on blank cards and included in the voting exercise. In the second phase of each cycle, *yuva saathi*s used stories based on prioritised problems to help groups discuss the causes of problems identified in the first phase. Stories prompted groups to consider causes at the family, community and societal levels. Groups decided which of these causes they would like to address and develop appropriate strategies. In the third phase, groups implemented their chosen strategies and explored some of the problems that were not prioritised but are considered relevant in light of the formative research. In the final phase, groups review their strategies, any challenges they faced, and how these challenges were overcome. They also organised a community meeting at the end of each PLA cycle, during which groups share their experience and learning and seek support from the wider community. Strategies implemented in earlier cycles continue to be implemented throughout the intervention implementation period. A further community meeting is held at the end of each of the four thematic PLA cycles, and an overall evaluation meeting to discuss all strategies implemented and the way forward was also held at the end of the intervention.

Additional intervention materials are available upon request from the corresponding author.

# S6. Revised sample size and power calculation following interruption due to the COVID-19 pandemic

Date: 28 May 2020

This document outlines the effects of stopping data collection due to COVID-19 on survey sample size and power to detect differences in primary outcomes. Stata code for sample size calculations are provided at the end of the document.

**Original Sample size calculation in statistical analysis plan**

The size of the study area was chosen for logistical reasons. With a district-level crude birth rate of around 23 per 1000 population and accounting for child deaths, we expected to find an estimated 8800 adolescents aged 10-19 (4400 girls) in our proposed intervention area. We anticipated that villages and their adjoined hamlets would have an average population of c.1000 each. In each village, there would be c.220 adolescent boys and girls aged 10-19 years.

3324 adolescent girls aged 10-19 years participated in the baseline survey: 82% of an estimated 4068 girls in the 38 clusters. The mean number of girls in each cluster was 87 (standard deviation 29.9). Assuming we are able to interview the same number of girls in the endline survey, with a mean cluster size of 87 and a coefficient of variation of cluster sizes of 0.3, the trial will have **80% power** to detect:

1. a nine percentage point increase in the proportion of girls attending school or college (ICC 0.03), from a baseline prevalence of 69% to 78%; **detectable difference 0.09**
2. a 0.9 point increase in mean dietary diversity score (ICC 0.40), from a mean baseline score of 3.4 (SD 1.4) to 4.3; **detectable difference 0.9**
3. a 2.7-point decrease in Brief Problem Monitor - Youth score (ICC 0.39) from a mean baseline score of 6.0 (SD: 4.3) to 3.4, all at a significance level of 0.05. **detectable difference 2.6**

We performed these calculations in Stata (version 14). We anticipate that including baseline data in our analysis will lead to gains in power but do not attempt to quantify these here.

**Situation April 2020**

Baseline data collection previously completed in 2016-17. Endline data collection interrupted in March 2020 at the point where 37% of the population (1478 participants) had been interviewed, with variability between clusters. Data were obtained from 29 clusters, 14 in one arm and 15 from the other.

**Note that 37% of the population corresponds to around 45% of the planned number of achieved interviews specified in the sample size calculation (total 38 x 87 = 3306)**

The mean cluster size is 51, and that the coefficient of variation of cluster size at endline is higher than at baseline, at 0.61 (31/ 51).

The original calculations are expressed in terms of detectable differences, because the sample size had effectively been determined before the calculation was made.

To assess the impact of stopping data collection now we have recalculated the detectable differences, with power and parameters as before. (Note we have taken the data as arising from 14 clusters per arm as the Stata command clustersampsi for sample size calculation in cluster trials assumes equal numbers per arm, this is slightly conservative).

1. **Detectable difference 0.12** (proportion increased from 69% to 81%)
2. **Detectable difference 1.15** (mean increased from 3.4 to 4.55)
3. **Detectable difference 3.49** (mean reduced from 6.0 to 2.51)

**Interpretation:**

1. The ICC for the first outcome is moderate, and for the second and third outcomes is very high. Given these ICCs the loss in power from collecting only 45% of the expected data is slight, but the loss of 9 clusters from the data collection is more important. Consequently the **detectable differences have increased by roughly a quarter**.
2. Recall that in the original calculation we do not take account of the increase in power due to the baseline data. If we actually are happy with the detectable differences in the original calculation, then we may expect these differences remain detectable, as the **loss in power from the reduced endline data collection is likely offset by the baseline data**.

**Stata code for sample size calculations**

*original (for education, dietary diversity and mental health)

clustersampsi, binomial detectabledifference p1(0.69) m(78) k(19) rho(0.03) size_cv(0.3)

clustersampsi, detectabledifference mu1(3.4) sd1(1.4) m(78) k(19) rho(0.4) size_cv(0.3)

clustersampsi, detectabledifference mu1(6) sd1(4.3) m(78) k(19) rho(0.39) size_cv(0.3)

* reduced data (for education, dietary diversity and mental health)

clustersampsi, binomial detectabledifference p1(0.69) m(53) k(14) rho(0.03) size_cv(0.61)

clustersampsi, detectabledifference mu1(3.4) sd1(1.4) m(53) k(14) rho(0.4) size_cv(0.61)

clustersampsi, detectabledifference mu1(6) sd1(4.3) m(53) k(14) rho(0.39) size_cv(0.61)

# S7. Comparison of baseline information for full and reduced samples followed up at endline

**Table S7: Baseline characteristics of full and reduced samples followed up at endline**

|  | **Baseline** | | | | **Endline** | |
| --- | --- | --- | --- | --- | --- | --- |
|  | **Participants in all 38 clusters (n=3324)** | | **Participants in 29 clusters followed up at endline (n=2449)** | | **Participant characteristics in endline survey (n=1478)** | |
| **Characteristic** | **Control** | **Intervention** | **Control** | **Intervention** | **Control** | **Intervention** |
| Village | 73 | 70 | 75 | 74 | 78 | 80 |
| Tribal | 87 | 77 | 85 | 76 | 84 | 75 |
| Sarna religion | 87 | 76 | 86 | 75 | 82 | 68 |
| Married adolescent | 11 | 10 | 11 | 10 | 5 | 5 |
| Personal mobile phone | 15 | 16 | 15 | 14 | 16 | 17 |
| Wealth quintile |  |  |  |  |  |  |
| *Highest* | 17 | 22 | 16 | 21 | 30 | 20 |
| *Second highest* | 19 | 23 | 20 | 23 | 17 | 15 |
| *Middle* | 19 | 19 | 18 | 17 | 20 | 30 |
| *Second lowest* | 22 | 20 | 22 | 21 | 16 | 20 |
| *Lowest* | 23 | 17 | 25 | 18 | 16 | 24 |
|  |  |  |  |  |  |  |
| Daily paid labour (MNREGA) | 39 | 45 | 39 | 49 | 24 | 16 |
| Toilet (use field) | 93 | 90 | 91 | 95 | 49 | 49 |
| Age group (15-19 years) | 49 | 44 | 48 | 44 | 46 | 42 |

# S8. Baseline characteristics by study arm

**Table S8: Baseline characteristics**

| **Characteristic** | **Control** | **Intervention** | **All** |
| --- | --- | --- | --- |
| Adolescent girls interviewed at baseline | 1690 (51%) | 1634 (49%) | 3324 |
| Household location |  |  |  |
| Main village | 1237 (73%) | 1137 (70%) | 2374 (71%) |
| Hamlet | 453 (27%) | 497 (30%) | 950 (29%) |
| Class or caste status |  |  |  |
| Scheduled tribe | 1469 (87%) | 1262 (77%) | 2731 (82%) |
| Scheduled caste | 2 (0.1%) | 13 (1%) | 14 (0.5%) |
| Other backward caste | 219 (13%) | 359 (22%) | 578 (17%) |
| Tribe (n=2731) |  |  |  |
| n | 1469 | 1262 | 2731 |
| Ho | 1464 (99%) | 1239 (98%) | 2703 (98%) |
| Santhal | 0 (0%) | 10 (1%) | 10 (0.4%) |
| Oraon | 0 (0%) | 1 (0.1%) | 1 (0.04%) |
| Munda | 4 (0.3%) | 12 (1%) | 16 (1%) |
| Other | 1 (0.1) | 0 (0%) | 1 (0.1%) |
| Religion |  |  |  |
| Sarna | 1464 (87%) | 1249 (76%) | 2713 (82%) |
| Hindu | 189 (11.1%) | 280 (17%) | 469 (14%) |
| Christian | 17 (1%) | 82 (5%) | 99 (3%) |
| Other | 20 (1%) | 23 (1%) | 43 (1%) |
| Adolescent’s marital status |  |  |  |
| Not married | 1498 (89%) | 1475 (90%) | 2973 (89%) |
| Married | 192 (11%) | 159 (10%) | 351 (11%) |
| Literacy |  |  |  |
| Cannot read | 331 (20%) | 276 (17%) | 607 (18%) |
| Reads with difficulty | 538 (32%) | 450 (28%) | 988 (30%) |
| Reads easily | 821 (49%) | 908 (55%) | 1729 (52%) |
| Personal mobile phone ownership | 248 (15%) | 255 (16%) | 503 (15%) |
| Household maintains small kitchen garden | 657 (39%) | 953 (58%) | 1610 (49%) |
| Multidimensional poverty quintile |  |  |  |
| *Highest (least poor)* | 287 (17%) | 364 (22%) | 651 (20%) |
| *Second highest* | 327 (19%) | 374 (23%) | 701 (21%) |
| *Middle* | 318 (19%) | 306 (19%) | 624 (19%) |
| *Second lowest* | 367 (22%) | 319 (20%) | 686 (21%) |
| *Lowest (poorest)* | 391 (23%) | 271 (17%) | 662 (20%) |
| Daily paid labour (MNREGA) | 660 (39%) | 739 (45%) | 1399 (42%) |
| Toilet |  |  |  |
| Not improved | 41 (2%) | 49 (3%) | 90 (3%) |
| Improved | 79 (5%) | 108 (7%) | 187 (6%) |
| Field | 1570 (93%) | 1477 (90%) | 3047 (92%) |
| Age group |  |  |  |
| 10-14 years | 869 (51%) | 913 (56%) | 1782 (54%) |
| 15-19 years | 821 (49%) | 721 (44%) | 1542 (46%) |
|  |  |  |  |
